# Towards a Cardoon (*Cynara cardunculus* var. *altilis*)-Based Biorefinery: A Case Study of Improved Cell Cultures via Genetic Modulation of the Phenylpropanoid Pathway

**DOI:** 10.3390/ijms222111978

**Published:** 2021-11-05

**Authors:** Dario Paolo, Franca Locatelli, Eleonora Cominelli, Raul Pirona, Sara Pozzo, Giulia Graziani, Alberto Ritieni, Monica De Palma, Teresa Docimo, Marina Tucci, Francesca Sparvoli

**Affiliations:** 1 National Research Council—Institute of Agricultural Biology and Biotechnology (CNR-IBBA), Via Edoardo Bassini 15, 20133 Milano, Italy; locatelli@ibba.cnr.it (F.L.); cominelli@ibba.cnr.it (E.C.); pirona@ibba.cnr.it (R.P.); sara.pozzo@ibba.cnr.it (S.P.); 2 Department of Pharmacy—University of Naples Federico II (UNINA), Via Domenico Montesano 49, 80131 Naples, Italy; giulia.graziani@unina.it (G.G.); alberto.ritieni@unina.it (A.R.); 3 National Research Council—Institute of Bioscience and Bioresources (CNR-IBBR), Via Università 133, 80055 Portici, Italy; monica.depalma@ibbr.cnr.it (M.D.P.); teresa.docimo@ibbr.cnr.it (T.D.); marina.tucci@ibbr.cnr.it (M.T.)

**Keywords:** plant cell cultures, lignin, cellulose accessibility, nutraceuticals, MYB4

## Abstract

Cultivated cardoon (*Cynara cardunculus* var. *altilis* L.) is a promising candidate species for the development of plant cell cultures suitable for large-scale biomass production and recovery of nutraceuticals. We set up a protocol for *Agrobacterium tumefaciens*-mediated transformation, which can be used for the improvement of cardoon cell cultures in a frame of biorefinery. As high lignin content determines lower saccharification yields for the biomass, we opted for a biotechnological approach, with the purpose of reducing lignin content; we generated transgenic lines overexpressing the *Arabidopsis thaliana* MYB4 transcription factor, a known repressor of lignin/flavonoid biosynthesis. Here, we report a comprehensive characterization, including metabolic and transcriptomic analyses of *AtMYB4* overexpression cardoon lines, in comparison to wild type, underlining favorable traits for their use in biorefinery. Among these, the improved accessibility of the lignocellulosic biomass to degrading enzymes due to depletion of lignin content, the unexpected increased growth rates, and the valuable nutraceutical profiles, in particular for hydroxycinnamic/caffeoylquinic and fatty acids profiles.

## 1. Introduction

Among the family of Asteraceae, *Cynara cardunculus* L. (2n = 2x = 34) is a species of perennial herbaceous plants with annual growth cycle; the species consists of three closely related botanical varieties: the wild cardoon (var. *sylvestris*), the globe artichoke (var. *scolymus*), and the cultivated cardoon (var. *altilis*) [1]. Given the full cross-compatibility between these varieties, in addition to phenotypic and genetic evidences, previous studies concluded that both the cultivated cardoon and the globe artichoke were domesticated from wild cardoons [2,3]. Cultivated cardoons are well-adapted to the semi-arid environment of the Mediterranean basin, where they are considered a traditional crop. Cardoons are in fact grown as vegetables in southern Europe and north Africa, and consumed for their whitened fleshy stalks; flowers are harvested and used as vegetable rennet [4]. 

Over the last 30 years, the potential of cultivated cardoon for biomass production has been increasingly investigated [5,6]. Advantages are represented by its perennial nature, the ability to photosynthesize during winter, and the high yields of lignocellulosic biomass (approximately 5–30 t/ha/year of dry matter, reviewed by [7]) obtained with minimal agricultural inputs. Moreover, cardoon is a highly adaptive species able to tolerate abiotic stresses such as soil salinity [8,9], drought [10], and soil contamination by heavy metals [11,12].

The use of cardoon to produce energy has been thoroughly evaluated [13,14,15,16]; a recent study reported that the total energy (E) obtainable from cardoon biomass was 236 GJ/ha/year, with an increase of E measured over the three cropping years of the experiment, suggesting that the use of this species as an energy-crop might be effective especially in semi-arid areas with low productivity soils [17]. Cardoon stalks have also been considered as a good source of fiber for the production of paper [18]. 

Cardoon biomass should also be considered for the high content in specialized metabolites (SMs); these molecules are in fact high-value compounds in a biorefinery perspective. Notably, cardoon extracts, especially from leaves, are rich in phenylpropanoids [19]. The most representative extracts are hydroxycinnamic and caffeoylquinic acids (particularly chlorogenic acid, CGA), *p*-coumaric acid, and several flavonoids (vitexin, luteolin, apigenin, naringin) [20,21,22]; these molecules mainly contribute to the antioxidant properties of the cardoon extracts [23,24].

For example, CGA has been described as a bioactive molecule against obesity, diabetes, cancer, and for its role in cardio and neuro-protection [25]. Moreover, cardoon extracts have potential in the prevention of hepatic and cardiac oxidative stresses [26,27], and they have been studied for their anti-microbial and anti-fungal activities related to the presence of flavonoids [24,28]. Therefore, the recovery of such nutraceuticals would be impactful for their possible downstream uses.

The importance of profiling phenylpropanoids is noteworthy when it comes to lignin. The term lignin refers to a large class of structural phenolic polymers that create complexes with the polysaccharides of cell walls (cellulose, hemicellulose and pectins); the abundance and chemical nature of such complexes contribute to the recalcitrance to the microbial or enzymatic degradative process of lignocellulosic biomass [29]. Therefore, chemical/physical or biological pre-treatments are required to improve the digestibility and availability of the cellulose fraction to enzymatic degradation, and facilitate the recovery of fermentable sugars (saccharification). However, such pre-treatments are expensive and invasive, and they determine a reduction in the sustainability of biomass valorization [30]. Consequently, using plant biomass with reduced and/or easily removable lignin is advantageous, and efforts have been performed to reach this goal through biotechnological approaches [31]. 

Another interesting feature of cardoon biomass is represented by its oil content (largely derived from seeds); cardoon oils, given their high unsaturated to saturated fatty acids ratio, the high levels of oleic and linoleic acids, and stability to oxidation [22,32], have potential for nutraceutical use, lowering serum cholesterol levels [33], and for biorefinery, e.g. for the production of bioplastics [34]. Indeed, plant oils and fats are among the most important renewable raw materials of the chemical industry, and examples of industrial applications include their use in environmentally friendly fluids and lubricants [35]. More recently, the possibility of functionalizing fatty acids skeletons through organic synthesis and/or microbial reactions gave strong emphasis to the possibility of improving oil production in plants [36]. Consequently, modification of the fatty acid flux, especially in favor of high monounsaturated fatty acids (MUFAs) accumulation, represents a major goal for biotechnology oriented towards green chemistry.

Among the drawbacks of cardoon biomass production from field cultivation, there are the variability in yields [37] and in the quality of the lignocellulosic biomass [38]. Field production also affects SMs: most of the cardoon genotypes assessed suffer from strong environmental effects on their phenolic profiles [39], and, in particular, alterations of the phenylpropanoid levels can be traced back to biotic and abiotic stressors [40]. Similarly, differences in the fatty acid profile of oils have been described as a result of genotype, plant tissues, climatic factors, and geographical regions [22,32,41,42]. For these reasons, the development of cardoon cell cultures for biorefinery applications is a promising strategy alternative to field production. Undifferentiated plant cell cultures (PCC) are totipotent, i.e., able to express the full genetic machinery of their species/tissue of origin. Characterization and improvement of PCC is thus an important target of research, to overcome limitations imposed by climatic changes and/or pathogen attacks, and by the genetic segregation of traits of interest that are cancelled with the propagation of selected stable cell lines of clones [43,44]. Moreover, many nutraceuticals are usually accumulated in low concentration in plant organs; the use of PCC and bioreactors, coupled with the possibility of selecting the best lines (and modifying their genome) and with elicitation techniques, improves the economic gains derived from the production, while at the same time reducing the environmental impacts bound to field cultivation [45,46]. Furthermore, PCC are devoid from the risk of containing toxic substances, such as residual pesticides. Altogether, this paves the way to the development of a pipeline of great interest to use PCC in the frame of biorefinery [44,47]. 

In this study, we tested the potential of improving cardoon PCC for biorefinery. To this end, we developed a method for the stable genetic transformation of cardoon cells via *Agrobacterium tumefaciens*, with the goal to reduce lignin biosynthesis. Given the challenges to single out an enzyme solely responsible of a complex metabolic flux (as for the phenylpropanoid pathway), the use of transcription factors (TFs) has revealed a successful approach to modify the biosynthesis of SMs [43,44]. As largely investigated, MYB-TFs, among their role in numerous physiological pathways, are responsible for the regulation of the biosynthesis of phenylpropanoids; in this regard, functional characterization approaches in different species indicated that MYBs acting as transcriptional repressors have more extensive effects than the corresponding MYB activators (recently reviewed by [48]). 

Hence, we overexpressed in cardoon cell cultures AtMYB4, an R2R3-type TF of the MYB family (subgroup 4), a known transcriptional repressor of lignin biosynthesis in *Arabidopsis thaliana*. This TF was initially characterized as a repressor of the sinapate ester biosynthesis in response to UV-B [49]. The activity of transcriptional repression of the lignin pathway is highly conserved among MYB4 orthologues in plants [50]: a reduced lignification is in fact obtained from the heterologous expression of the maize (*Zea mays*) *ZmMYB31* and *ZmMYB42* genes in Arabidopsis [51,52]; the same phenotype was described in tobacco plants overexpressing the wheat (*Triticum aestivum*) *TaMYB1D* and *TaMYB4* [53,54], and the switchgrass (*Panicum virgatum*) *PvMYB4* genes [55]. In poplar (*Populus trichocarpa*), the overexpression of the *PtMYB156* gene negatively affects secondary cell wall biosynthesis [56], while the loss-of-function of a rice (*Oryza sativa*) MYB4 transcription factor (*Os*MYB108) favors lignification [57]. In addition, MYB4 TFs were also suggested to be repressors of flavonoid biosynthesis [58,59]. Only very recently have the detailed molecular mechanisms of this additive MYB4 function been fully elucidated: MYB4 can repress the transcription of *ADT6* gene catalyzing the final step of biosynthesis of phenylalanine (the precursor of flavonoid biosynthesis), and can also attenuate the transcriptional function of the MYB-bHLH-WDR complexes to regulate anthocyanin and proanthocyanidin biosynthesis. It has been hypothesized that AtMYB4 may function as a sensor in the phenylpropanoid pathway regulating the cross-talk between primary (phenylalanine) and secondary metabolism [60]. Moreover, since our investigation is also addressed towards the development of cardoon cell lines to be used for the production of fatty acids, we also investigated the effects of *AtMYB4* overexpression on the fatty acid profile of the transgenic lines.

For these reasons, we obtained independent *AtMYB4*-overexpressing transgenic lines, exploring their potential value in the frame of biorefinery; we therefore analyzed their growth curves, lignin content, and accessibility to degradation of the cellulose fraction, in addition to characterizing their biochemical profile, both for the phenolic and fatty acid profiles. 

## 2. Results

### 2.1. Development of a Method for the Stable Transformation of Cardoon

**Figure 1:**
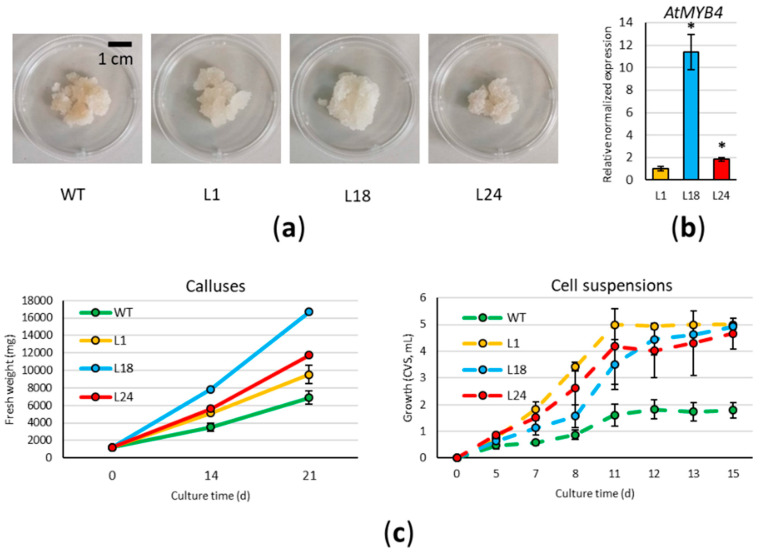
Phenotype and growth of cardoon lines: (**a**) Calluses of wild type (WT) and *AtMYBoe* lines (L1, L18, L24) grown for 21 days on solid medium; (**b**) Relative expression level (mean ± standard deviation, S.D.) of the transgene in the three lines selected for this study. Asterisks indicate significant differences (*p* < 0.01); (**c**) Growth curves for calluses grown on solid medium (left) and for cell suspension grown in liquid medium (right). CVS: cell volume after sedimentation. Each value represents the mean of three biological replicates ± S.D.

In this work, we developed a method for the stable genetic transformation of cardoon leaf-derived cell cultures via *A. tumefaciens*, overexpressing the coding sequence of the *A. thaliana* MYB4 transcription factor under the control of the constitutive cauliflower mosaic virus (*CaMV35S)* promoter. Five distinct transformation experiments were carried out: wild type (WT) calluses obtained from cardoon plants (“Spagnolo” genotype) were co-cultivated with AGL1 or EHA105 *A. tumefaciens* strains carrying the *p35S::AtMYB4* construct. To select transformants, the co-cultivated cardoon cells were spread on the selective solid Gamborg B5 medium containing 10 mg/L phosphinothricin (PPT) and 200 mg/L cefotaxime (CFX), sub-culturing emerging calluses to new medium every 21 days. While at first, we counted 48 independent calluses emerging from the selective plates, only 15 of them survived the second and further sub-culturing rounds. Three months after transformation, all 15 resistant calluses were checked via PCR, and confirmed to be carrying the transgene and devoid of *Agrobacterium* contamination. All lines were maintained on PPT and CFX-containing media in order to avoid genetic chimerism, and the three *AtMYB4oe* lines selected to be analyzed for this study are here referred as L1, L24 (from infection with the AGL1 strain), and L18 (from infection with the EHA105 strain). The three lines appeared similar to each other, but different from the WT, with the callus texture softer than WT and thus more easily spreadable when sub-cultured (Figure 1a). RT-qPCR was used to verify the expression of *AtMYB4*, showing that the different lines expressed the transgene at different levels, with L1 having the lowest and L18 the highest level of expression (Figure 1b).

### 2.2. AtMYB4oe Lines Have Higher Growth Rates Than Wild Type

Growth rates of WT and *AtMYB4oe* lines were monitored both on solid and liquid media (Figure 1c); for this purpose, the starting material belonged to the XXII round of sub-cultures for WT, and to the XIV for *AtMYB4oe* lines. Calluses (6 × 200 mg each) were placed on Gamborg B5 solid medium in Petri dishes, and the total cellular mass (fresh weight, FW) was evaluated at 14 and 21 days of the sub-culture. All transgenic lines showed a significant higher growth rate than WT at both time points; in particular, after 14 days, *AtMYB4oe* biomass was 1.5-fold (L1 and L24) and 2-fold (L18) higher than WT. A similar trend was observed after 21 days in L1 and L24, whereas L18 mass increased 2.4-fold with respect to the WT. The fastest growing lines (L18 and L24) were also characterized by the lowest variation among experimental replicates, granting a more uniform growing behavior compared to WT. Relative differences of FW observed between WT and *AtMYB4oe* lines were confirmed in the comparison of dry weights (DW), as the percentage of the ratio between DW and FW was nearly identical for all samples (3.6%). Cell suspensions were grown for a maximum of 15 days, and the cell volume after sedimentation (CVS) was used to follow their growth. During the initial lag phase up to the fifth day, we observed a similar growth in all lines, whereas during the exponential phase, from the 5th to the 11th day, all the transgenic lines had a higher growth rate than the WT one (biomass 2–3-fold higher). L18 and L24 showed a slightly lower growth rate than L1 at the end of the exponential phase; nevertheless, on the 15th day, all the transgenic lines reached a biomass 3-fold higher than the WT. 

Additionally, as plants of the “Spagnolo” genotype are tolerant to salinity stress [9], we grew WT and transgenic lines, adding 100 mM NaCl to solid medium. WT line showed some degree of tolerance to this condition, whereas *AtMYB4oe* lines did not grow, and turned quickly to necrosis (Figure S1).

### 2.3. AtMYB4oe Lines Show Decreased Phenolic Compounds and Lignin Content and Enhanced Enzymatic Saccharification Efficiency

**Figure 2:**
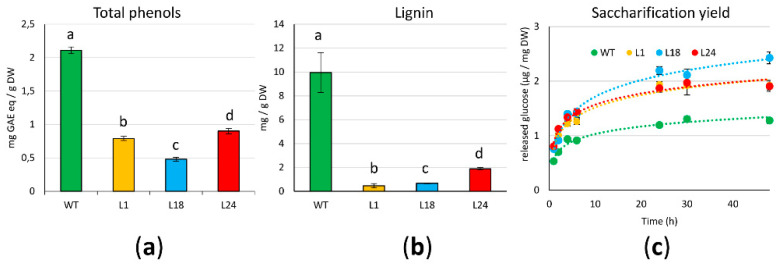
Phenols/lignin content and saccharification yields of 21 days-old calluses: (**a**) Folin–Ciocalteu’s quantification of total phenols, expressed as mg of GAE (Gallic Acid Equivalents)/g dry weight (DW) ± S.D; (**b**) quantification of lignin via thioglycolic acid (TGA) derivatization. Bars indicate means ± S.D. of three biological replicates. Different letters above bars indicate statistically significant differences (*p* < 0.01); (**c**) saccharification yields expressed as mean values of µg released glucose/mg DW ± S.D.

To investigate if the overexpression of *AtMYB4* exerted a similar repression on the phenylpropanoid pathway to what was observed in other species, particularly on lignin, and as recently demonstrated also on anthocyanins and proanthocyanidins [60], we measured the amount of phenolic compounds in extracts of 21 days-old freeze-dried calluses (sub-cultures: XXII for WT and XIV for *AtMYB4oe* lines). Figure 2a shows that all *AtMYB4oe* lines have significantly less phenolic compounds than WT, with the strongest reduction in L18. As lignin monomers are one of the main products of the phenylpropanoid pathway, we quantified lignin content. As for total phenolic compounds, we reported a significant lignin reduction (close to −90%) for all transgenic lines (Figure 2b).

Next, we wanted to test if the depletion in lignin content of *AtMYB4oe* lines had an impact on the saccharification yields of the lignocellulosic biomass, measured as the amount of fermentable sugars obtained from the hydrolysis of the cell wall polysaccharides. This provides an estimate of the accessibility of the cell wall to degradative enzymes and, consequently, of the accessibility of biomass to extract useful compounds. To do this, we measured over time the amount of glucose released from the freeze-dried biomass subjected to enzymatic degradation with a cellulase (Figure 2c). Over 48 h of reactions, logarithmic regression models fit very well with saccharification trends for WT and transgenic lines (R^2^ > 0.97); the amount of released glucose from *AtMYB4oe* lines was higher than WT already at the earliest time points of the reactions (1 h, 2 h), and this was also confirmed when the reaction plateau was reached. In fact, from 24 h on, L1 and L24 had a 1.3-fold increase of released glucose over WT, a value that is even higher (2-fold) in L18. 

### 2.4. AtMYB4oe Lines Show a Different Profile of Polyphenolic Compounds and Fatty Acids Compared to Wild Type

WT and transgenic lines were analyzed for the characterization of phenolic compounds, antioxidant activities, and fatty acids (Table 1). As for the other analyses, measurements were performed on 21-days old calluses (sub-cultures: XXII for WT, and XIV for *AtMYB4oe* lines). Typical full-scan chromatograms and the specifications of the observed peaks are reported in Figure S2. 

On average, WT lines have a total polyphenolic content of 6962 µg/g DW, while transgenic lines are characterized by a significant reduction, strongest for L18 (−96%). In all lines, hydroxycinnamic acids account for the largest fraction; phenolic acids are present in significantly lower quantities and, among them, *p*-coumaric content is 3.50 µg/g DW in WT, reduced only in L18 (−57%). Eight peaks were assigned to non-anthocyanin flavonoids: quercetin and the deriving quercetin-glucoside, kaempferol and kaempferol-glucoside, naringin, luteolin, myricetin, and apigenin, all of which resulted reduced in L18. Among nutraceuticals, CGA (319 µg/g DW in WT) was reduced in L18 compared to WT (−69%); notably, the opposite is true for L1 and L24, which accumulated CGA more than WT (+60% and +32%, respectively). Furthermore, we observed that the reduction in phenolics of *AtMYB4oe* lines was also reflected in a reduced antioxidant activity, as evaluated with three different techniques: 2,2-diphenyl-1-picrylhydrazyl (DPPH) radical scavenging, 2,2′-azino-bis (3-ethylbenzothiazoline-6-sulfonic acid) (ABTS) decolorization, and Ferric Reducing Antioxidant Power (FRAP) assays. The correlation between phenol content and antioxidant activity is high for all the three assays: R^2^DPPH = 0.96, R^2^ABTS = 0.99, and R^2^ABTS = 0.87. 

Considering the interest of the fatty acids profile typical of cardoon oil, we also characterized this fraction. The oil content of the biomass is slightly reduced in transgenic lines, with L24 exhibiting the lowest oil yield at 7.2%. We characterized, in detail, the fatty acid fraction by means of gas chromatography analysis (Table 1). Among saturated fatty acids (SFAs), palmitic acid (C16:0) is the most abundant, followed by stearic acid (C18:0), and lignoceric acid (C24:0); for all of these SFAs, no significant changes between WT and transgenic lines were detected. The content of MUFAs is mostly represented by oleic acid (C18:1 n9), which accounts for 3.4% of total fatty acids in WT and L18; interestingly, we detected a 3–4-fold increase in L1 and L24 lines. The content of PUFAs consists mostly of linoleic acid (C18:2 n6, LA) and linolenic acid (C18:3 n3, ALA), whose levels behave oppositely in WT and transgenic lines; notably, the content of LA in *AtMYB4oe* lines represents a 2-fold increase over WT. On the other hand, WT content of ALA is reduced by 2–4 fold in transgenic lines. Overall, there are no significant changes compared to WT in the total unsaturated:saturated fatty acids ratio, which ranges from 2.4 to 2.6 in transgenic lines; L1 and L24 transgenic lines, however, differ significantly in the PUFA:MUFA ratio from WT and L18 line (5.3 and 4.3 vs. 14.7 and 15.4, respectively).

### 2.5. Transcriptomic Analysis of AtMYB4oe Lines

Transcriptomic changes induced by the heterologous overexpression of *AtMYB4* were evaluated by RNA-seq analysis. The dataset included 12 samples divided in the four experimental groups (WT—XXII sub-culture—as a control and three independent transgenic lines: L1, L18, and L24—XIV sub-culture). The quality of the raw reads was very high. After trimming, an average of 91.6% of reads (17.4 M reads/sample) were retained and usable for downstream analyses. All samples presented over 94% of unique mapping to the reference genome, with very low percentages of multi-and un-mapped reads (Table S1). In order to quantify gene expression, fragments per kilobase of exon model per million bases mapped (FPKM) values were calculated. The overall quality of the experiment was then evaluated; on the basis of the high similarity between replicates, based on the Principal Component Analysis (PCA) using the normalized gene expression values as input (Figure S3a), data from all 12 samples were used for further investigations.

**Figure 3:**
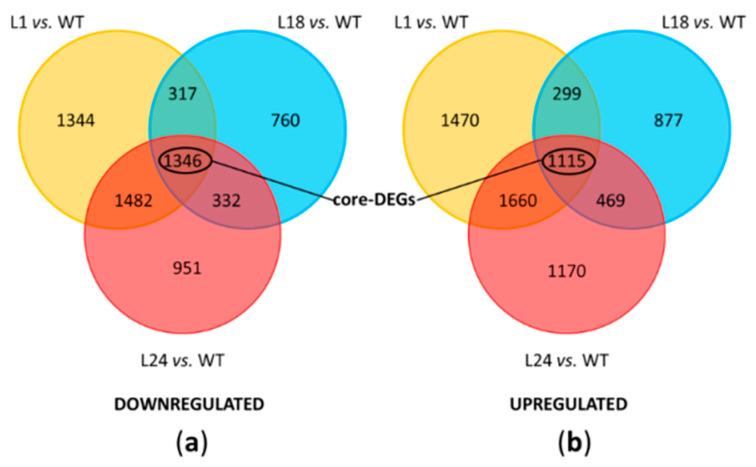
Differentially expressed genes (DEGs) between *AtMYB4oe* lines and WT: Venn diagrams of the number of (**a**) downregulated and (**b**) upregulated genes for all comparisons (FDR < 0.05).

A differential expression analysis was performed to identify the differentially expressed genes (DEGs) emerging from the three comparisons (L1 vs. WT, L18 vs. WT, and L24 vs. WT), as well as DEGs common to multiple comparisons. For each comparison, the relationship between fold change (FC) values and the statistical significance of DEGs was visualized via volcano plots, while MA-plots were used to identify normalization issues and expression-dependent patterns in the log ratios (Figure S3b,c). Each comparison produced several thousand DEGs (Figure 3). The transgenic line L1 was the one with the most DEGs, in comparison to WT (9033, downregulated = 4489/upregulated = 4544), followed by L24 (8225, downregulated = 4111/upregulated = 4114), and L18 (5515, downregulated = 2755/upregulated = 2760). In order to identify common pattern of gene expression among transgenic lines, we also considered the list of all the significant DEGs that overlapped among the three comparisons, which resulted in total of 2461 core-DEGs (downregulated = 1346/upregulated = 1115). Among core-DEGs, 632 had a log_2_FC < −1 and 579 had a log_2_FC > +1. The complete list of the identified DEGs for each comparison is provided in Table S2.

Validation of the RNA-seq expression data was performed by means of RT-qPCR, testing the expression of selected core-DEGs. In particular, in order to span a consistent range of FC values (−7.99 < log_2_FC < +8.14) and gene functions, the following were tested: genes encoding phenylpropanoid enzymes (*Ccrd_010165*/C4H, *Ccrd_015556*/4CL, *Ccrd_015561*/HCT, *Ccrd_004659*/CCoAOMT), other enzymes (*Ccrd_024577*/NADPH dehydrogenase, *Ccrd_000418*/xyloglucan endoglucosylase), cyclin-dependent kinase inhibitors (*Ccrd_010818*/KRP7-like), and TFs (*Ccrd_019107*/MADS-TF, *Ccrd_016332*/AP2-TF). The quality of the expression data from RNA-seq was excellent given the high correlation of expression values via validation (R^2^ = 0.9), and the consistency among biological and technical replicates analyzed (Figure S4).

### 2.6. Gene Ontology Enrichment Analysis (GOEA)

**Figure 4:**
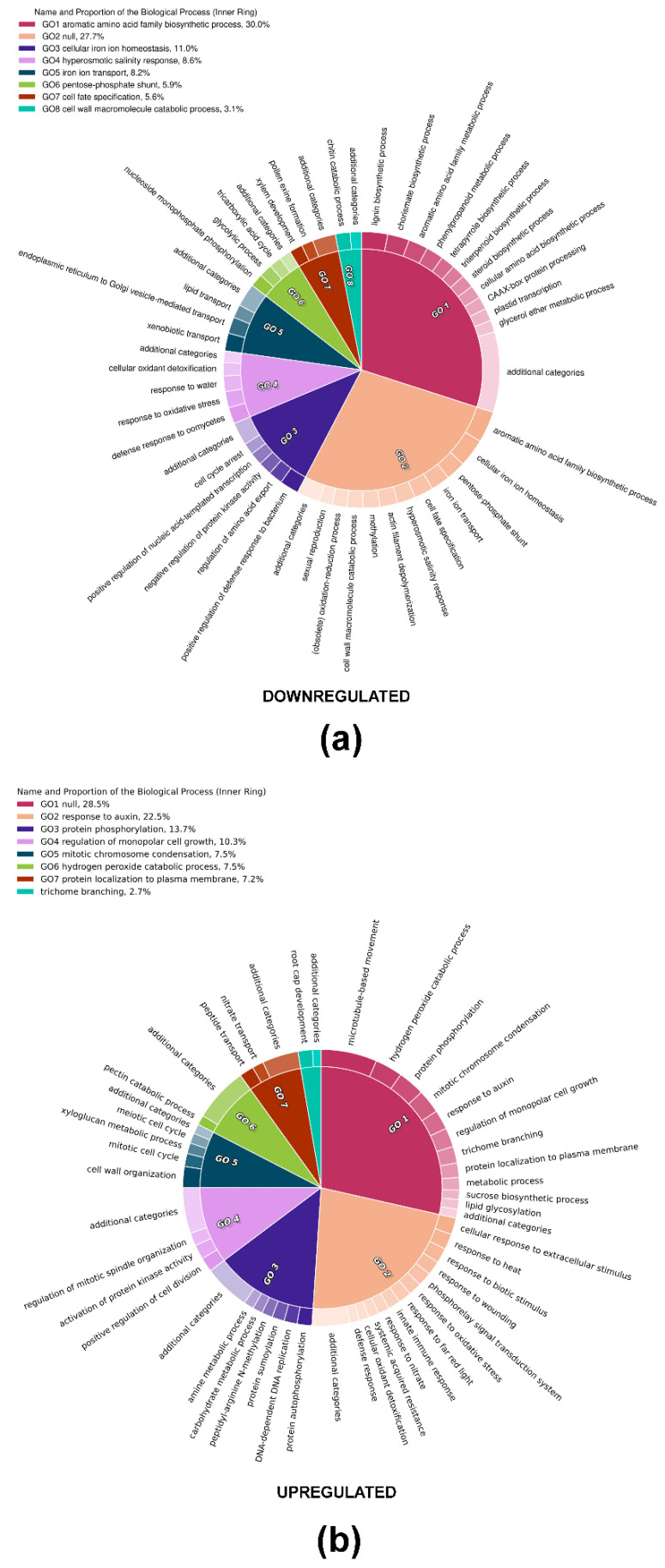
Gene Ontology Expression Analysis: Gene Ontology (**a**) downregulated and (**b**) upregulated terms referred to biological processes enriched among core-DEGs.

A Gene Ontology (GO), followed by a Gene Ontology Enrichment Analysis (GOEA), was performed with the core-DEGs to identify the most enriched GO terms. The complexity of the list of enriched GO terms for biological processes (BP), molecular functions (MF), and cellular components (CC) was reduced via the software REVIGO, in order to identify and collapse redundant terms. Figure 4 shows CirGO visualization of enriched BP; interestingly downregulated core-DEGs were categorized in processes such as lignin biosynthesis (GO:0009809), phenylpropanoid metabolism (GO:0009698), aromatic amino acids biosynthesis (GO:0009073), hyperosmotic salinity response (GO:0042538), and cell cycle arrest (GO:0007050). On the other hand, BP relative to upregulated core-DEGs included the mitotic cell cycle (GO:0000278), positive regulation of cell division (GO:0051781), mitotic chromosome condensation (GO:0007076), and microtubule-based movement (GO:0007018). For a complete exploration of GO annotations of core-DEGs, full GOEA lists are provided as Table S3.

Besides MYB4, the regulation of phenylpropanoid metabolism depends on many other transcription factors (TFs). Analysis of our transcriptomic data against the PlantTFDB database detected 153 genes encoding DE TFs (78 downregulated and 75 upregulated in *AtMYB4oe* lines, compared to WT), accounting for 6.2% of the total core-DEGs (Table 2). The most numerous TF family was bHLH (18), followed by NAC (17) and WRKY (15). A total of 11 core-DEGs encoded MYB-related TFs, here listed alongside their putative Arabidopsis paralogues: among these, six were downregulated (*Ccrd_007105*/*AtMYB5, Ccrd_006948*/*AtMYB79, Ccrd_002014*/*AtMYB15, Ccrd_001286*/*AtMYB19, Ccrd_018110*/*AtMYB48, Ccrd_013908*/*AtMYB107*), and five were upregulated (*Ccrd_002100*/*AtMYBS2, Ccrd_001632*/*AtMYB62, Ccrd_012299*/*AtMYB36, Ccrd_012123*/*AtMYB38, Ccrd_009207*/*AtMYB14*). Other TFs, such as bZIP, GRAS, AP2 and MADS, HSF, and bZIP, were also identified.

**Figure 5:**
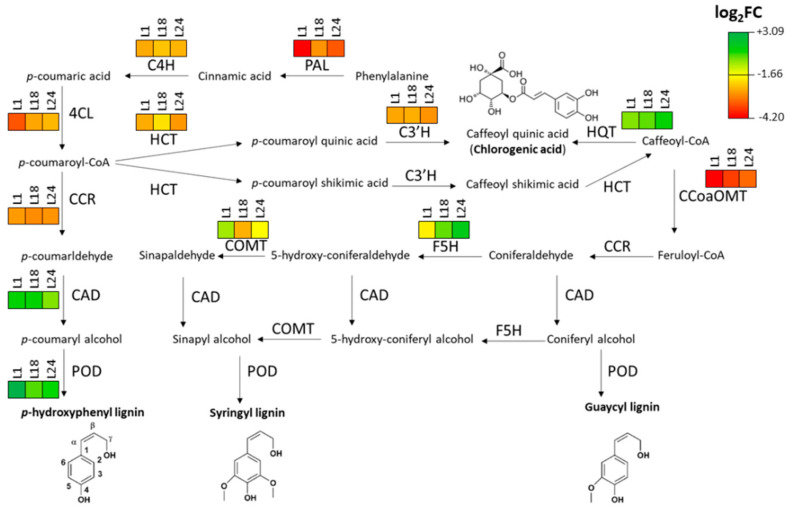
Biosynthetic pathway of phenylpropanoids and average expression levels of the relative genes in *AtMYB4oe* lines vs. WT.

Considering the observed reduction in the phenolic content of transgenic lines and the GOEA identifying several DEGs involved in phenylpropanoid pathways, as well as the changes in the fatty acids profile (particularly oleic acid), we focused our attention on the transcriptional changes that the *AtMYB4* overexpression induces on the biosynthesis of these compounds. Based on the Kyoto Encyclopedia of Genes and Genomes (KEGG) annotations of the phenylpropanoid biosynthesis (ccav:00940), we identified all *Cynara* enzymes involved in all the catalytic steps of the pathway leading to lignin monomers and CGA, and their relative genes among DEGs (Table S4). Similarly, we identified the main genes putatively involved in the production and conversion of oleic acid, as part of the biosynthesis of unsaturated fatty acids (ccav:01040) (Table S5). For the phenylpropanoid biosynthesis, the 12 main catalytic steps, identified by their Enzyme Commission (E.C.) number, involved a total of 73 genes found in at least one of the three DEG lists; the three transgenic lines had a similar number of DEGs belonging to this pathway: in L1, 55 DEGs (24 downregulated and 31 upregulated), in L18, 49 DEGs (27 downregulated and 22 upregulated), in L24, 58 DEGs (29 downregulated and 27 upregulated). Strong downregulation is particularly represented upstream in the pathway, as demonstrated by the number of core-DEGs encoding catalytic activities for the early biosynthetic steps: phenylalanine ammonia-lyase/PAL [E.C. 4.3.1.24], cinnamate 4-hydroxylase/C4H [E.C. 1.14.14.91], 4-coumaroyl CoA-ligase/4CL [E.C. 6.2.1.12], and hydroxyl-cinnamoyl-transferase/HCT [E.C. 2.3.1.133], with two downregulated core-DEGs found for each of these steps. Among later biosynthetic steps, peroxidase activity/POD [E.C. 1.11.1.7] accounted for the majority of the DEGs (41), followed by cinnamyl-alcohol dehydrogenase/CAD [EC:1.1.1.195]. Overall, average downregulation was stronger in the early catalytic steps of the pathway (Figure 5).

**Figure 6:**
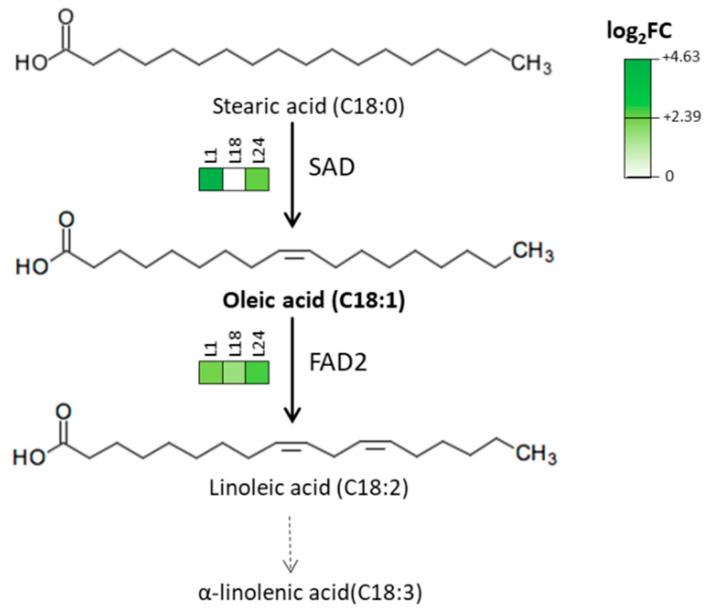
Biosynthesis of oleic acid and average expression levels of the relative genes in *AtMYB4oe* lines vs. WT.

When focusing on the biosynthesis of oleic acid (Figure 6), genes encoding the activity of soluble stearoyl-ACP desaturase/SAD [E.C. 1.14.19.2-1.14.19.11-1.14.19.26] activity were found to be upregulated in L1 and L24, while upregulated genes encoding the activity of ∆(12)-fatty-acid desaturase/FAD2 [E.C. 1.14.19.6-1.14.19.22] were found for all lines, including three upregulated core-DEGs.

## 3. Discussion

For this study, we set up a protocol for the stable genetic transformation of cardoon cell cultures, with the aim of lowering the lignin content of cell walls, and increasing the accessibility to degradative enzymes, by overexpressing the coding sequence of the Arabidopsis MYB4 transcription factor under the control of the constitutive *CaMV35S* promoter. While biolistic transformation of cell suspension from cardoon has been attempted before [61], to the best of our knowledge, ours is the first report of successful stable transformation of cardoon cell cultures via *A. tumefaciens*. 

To this end, we used two *Agrobacterium* strains (EHA105 and AGL1) with the same genetic background (C58), obtaining a similar number of transformation events, thus highlighting the reproducibility and value of this method.

The analyses of the transgenic lines proved that the *CaMV35S* promoter is suitable for overexpression strategies also in cardoon cells, as already shown for other Asteraceae such as artichoke [62], chicory (*Cichorium intybus* var. *sativum* L., [63]), and gerbera (*Gerbera jamesonii*, [64]). The three independent lines selected for this study overexpressed the transgene at significantly different levels, allowing for the exploration dose-dependent phenotypes. 

The overexpression of different MYB4 proteins resulted in reduced plant stature [49,59,65,66]. Moreover, it has been recently shown that in the dwarf *ref4-3* Arabidopsis plants, affected in a regulator of phenylpropanoid homeostasis [67], *AtMYB4* expression is up-regulated, and in the *myb4 ref4-3* double mutant there is a reversion to the wild type phenotype [60]. More generally, different lignin mutants exhibit impaired growth that interferes with the broader deployment of lignin engineered plants, a phenomenon referred to as lignin modification-induced dwarfism (LMID). Although the actual mechanism by which dwarfism arises remains unknown, some possible interpretations have been proposed [68]. Surprisingly, a remarkable and unforeseen characteristic of the cardoon *AtMYB4oe* lines is their faster and more replicable growth rate, in comparison to WT, both on solid and liquid media. L18 is the fastest line to accumulate biomass on solid medium, hinting at a correlation with its highest overexpression levels of *AtMYB4*. However, growth rates of all *AtMYB4oe* lines are of particular interest in the case of cell suspensions. The use of bioreactors for PCC depends in fact on the possibility of using appropriate liquid medium and apparatus to propagate cells [69]. Previous studies have already highlighted how cardoon cells are promising for the production of high yields of biomass [70], and for their use in continuous cell culture systems. This feature is due to the ability of these cells to adapt to new environmental conditions, and to the low aggregation and low cell growth on the walls of the fermenters [71]. Our transcriptomic data bolster the evidence of faster growth rates of *AtMYB4oe* lines: a significant number of upregulated DEGs was classified via GOEA as belonging to different activities related to mitosis. Among them, *Ccrd_006023* (orthologue of Arabidopsis *AUR3*) encodes a Ser/Thr kinase, whose activity peaks during cell division [72], *Ccrd_013806* encodes a DNA-polymerase and *Ccrd_000826* (orthologue of *AtHUB1*) positively regulates G2/M transition through histone monoubiquitination [73]. 

Several plant MYB TFs, in particular of R1R2R3-type, have been shown to regulate cell cycle [74], and this function has been reported also for the AtMYB59, AtMYB125, FOUR LIPS/AtMYB124, AtMYB88, AtMYB46, and AtMYB83 R2R3-type MYBs [74,75,76,77,78]. For AtMYB4 and its orthologues involvement in the control of cell cycle has not been reported; however, the reduced plant stature in different species overexpressing MYB4 orthologues, the faster growth rate of the *AtMYB4oe* cardoon lines, and the differences in their transcriptional modulation of cell cycle genes as compared with WT suggest a possible role of AtMYB4 in the regulation of cell cycle progression. A recent article characterized the MYB4-comprising regulatory network driving lignification in bamboo (*Phyllostachys edulis*), and the transcriptomic analyses confirmed that genes related to cell cycle activation and cell differentiation are downregulated as lignification progresses in shoots [79], supporting the idea of a negative correlation between the synthesis of lignin and growth rate. In addition to this, it was very recently shown that *AtMYB46* and *AtMYB83* in *Arabidopsis thaliana* and *RrMYB18* in *Rosa rugosa* are induced by wounding, and regulate the expression of genes involved in cell wall biosynthesis, including lignin, and cell cycle progression. Moreover, expression correlation analysis in different species revealed that this co-regulation of genes involved in both processes is highly conserved [74]. These results question the previous accepted suggestion that these two processes are independently regulated [80]. It can also be hypothesized that AtMYB4 may be part of this co-regulatory network, and that the opposite phenotype observed in plants and in PCC overexpressing MYB4 proteins might depend on the important and dispensable role of lignin in the two systems, respectively, and then in the different compensation mechanisms activated by the cells in plants compared to PCC.

A lack of growth of *AtMYB4oe* lines on 100 mM NaCl-medium is corroborated by the downregulation of hyperosmotic salinity response detected by GOEA, as well as by the reduction in antioxidant activity of all the transgenic lines; the increased salt-sensitivity could also be related to the reduction of lignin content in cell walls. On the contrary, WT tolerance to this stress condition confirms what was already revealed for plants of the same genotype [9].

We demonstrated that the levels of phenolic compounds were severely affected in *AtMYB4oe* lines. We also showed a high correlation between phenolics and antioxidant activities, coherent with previous studies on different cardoon tissues [22]. Folin–Ciocalteu’s quantification was used to quickly confirm the depletion of total phenolics, which accumulate at the lowest level in L18, another evidence of a putative dose-dependent effect of the overexpression of *AtMYB4*. 

We showed that lignin content was severely reduced in *AtMYB4oe* lines, and the depletion of total phenols and lignin was reflected by the RNA-seq data: GOEA of DEGs revealed the downregulation of genes involved in phenylpropanoid biosynthesis and, more specifically, in lignin biosynthesis and cell wall organization. Lignin polymers reinforce plant cell walls, binding cellulose fibers, and protecting them from enzymatic degradation. This evidently means that lignin is also a barrier to efficient biomass saccharification, thus making pretreatments of lignocellulosic biomass necessary to efficiently achieve the hydrolysis of polysaccharide fractions, for example, for the recovery of fermentable sugars [81]. We demonstrated that the reduction of lignin in *AtMYB4oe* lines significantly increases saccharification yields, as proven by the higher levels of released glucose: this is another fundamental and desirable feature for the use of these cell lines in bioreactors, as the reduction of lignin enhances the accessibility of the cellulose fraction to enzymatic degradation. A similar result was shown in planta through the overexpression of *PvMYB4* in switchgrass [66]. 

Altogether, the described biochemical and molecular evidence proves that the heterologous protein AtMYB4 also maintains its role when expressed in cardoon cells.

Notably, the overexpression of *AtMYB4* allowed for the simultaneous alteration of the expression of other TFs (including several other *MYBs*). This should be considered for the potential amplification effects on the modulation of the same pathway, or for cascade effects on other pathways. Amplification effects can for example be explained by the detected downregulation of *Ccrd_002014* (orthologue of *AtMYB15*), which is involved in lignin biosynthesis [82]. In a biorefinery context, in addition to the high yields of biomass, cardoon has been described for its rich phenolic profile, characterized by high-added value nutraceuticals [19,22]. Metabolic analyses revealed that cardoon calluses accumulate high levels of phenolics, in particular CGA and cynarine [22,83]. We confirmed the presence of high levels of total phenols in WT lines, that are even higher than previously observed in leaves of the same genotype [22], whereas total phenols were reduced in the *AtMYB4oe* lines, as expected. However, a deeper quantitative and qualitative characterization revealed interesting findings about specific nutraceuticals: CGA accumulates more in two of the lines we tested (L1 and L24) than WT. Similarly, in L1 and L24 lines, *p*-coumaric acid and flavonoids identified in the extracts (quercetin, kaempferol, naringin, luteolin, myricetin, and apigenin) accumulated at similar levels than WT. Notably, this confirms that while the overexpression of *AtMYB4* represses the phenylpropanoid pathway by reducing the production of lignin monomers, it does not necessarily depauperate nutraceuticals, which could be recovered from the cell cultures, increasing the interest in these lines from a biotechnological point of view. 

Given the potential of cardoon as a source of oils, we performed quantitative and qualitative analyses of the fatty acids profile of WT and transgenic lines. The use of friable calluses from other species has been previously suggested as a potential source for plant oils, especially for biofuels, such in the case of *Jatropha curcas* [84]; we have shown that leaf-derived cell cultures of cardoon only contain limited amounts of oil in WT (and even less in *AtMYB4oe* lines, though a more precise quantitative approach is required), a result that does not differ from what is observed in leaves, where the oil content ranges from 10 to 15%, depending on the genotype [22]. While the levels of calluses oil content do not match the average levels of seeds (25%, [22]), the faster accumulation of biomass of the transgenic lines could, however, offset this drawback. Unexpectedly, we have also observed how the overexpression of *AtMYB4* also reflects on the composition of oils. A major result is the 3-fold increase in the levels of oleic acid in two of the transgenic lines (L1 and L24), which should be taken in consideration both from a nutraceutical and industrial perspective. The increase of oleic acid is corroborated by the transcriptional data, as shown by the expression levels of genes encoding SAD activity, responsible for the conversion of saturated stearic acid to oleic acid [85]: only in L1 and L24 are SAD genes in fact upregulated, while their expression level does not change for L18, whose oleic acid levels do not differ from WT, despite being the line with the strongest AtMYB4 overexpression. High levels of oleic acid reduce oxidation of oils due to the presence of a single double bond in the C chain of the molecule—thus improving shelf life [86]; in addition to this, oleic acid is also beneficial to health as it inhibits fatty acid biosynthesis and cholesterogenesis in vivo as well as in vitro [33]. Moreover, we also report how the levels of linoleic acid increase in all transgenic lines, coherently with the strong upregulation of genes encoding FAD2 activity, responsible for the additional desaturation that converts oleic acid into linoleic acid [85]. From a nutritional perspective, it is also notable that the linoleic/linolenic acids ratio (omega-6/omega-3), which was 1:2 in WT, drastically changes in transgenic lines, with values ranging from 2–4:1, overlapping the range considered optimal for nutritional value of oils [87]. 

Many biotechnological approaches have been conducted to manipulate the fatty acid composition of oilseed crops, both for industrial and food uses [88]. This has been considered over the years an important goal of research, for example, for the increase of oleic acid levels; this has been attempted mainly via the alteration of genes encoding fatty acid biosynthetic enzymes [89]. For example, high oleic acid mutants defective in desaturase *FAD2* genes (and therefore impaired in the conversion of oleic into linoleic acid) have been generated and investigated in maize (*Zea mays,* [90]), soybean (*Glycine max,* [91]), and peanut (*Arachis hypogaea,* [92]). Indeed, all of these approaches envisaged the use of metabolic engineering strategies to make the whole plant a “platform” for the production of industrial lipids. To the best of our knowledge, ours is the first report of efficient modifications of fatty acid profiles in plant calluses, and of how *MYB4* levels could also affect the content and composition of plant oils; such a role is otherwise known for other MYB-TFs, that interestingly we found among downregulated genes of transgenic lines, as in the case of MYB5 and MYB107 [93,94]. Alternatively, the alterations observed in the fatty acid profiles of the described cell lines could be traced back to the growth characteristics of the *AtMYB4oe* lines, rather than directly to MYB4 activity. Studies on cell suspensions and calluses from *Acer pseudoplatanus* and *Arabidopsis thaliana* have in fact highlighted how faster growth rates correlate with higher levels of oleic acid (C18:1), and the decrease in the levels of PUFAs (particularly C18:2 and C18:3), a phenomenon that might be explained by considering that when membrane synthesis/turnover are fast, the activities of FAD2/FAD3 might to match phospholipid synthesis and editing, leading to 18:1 accumulation [95]; interestingly, the same positive correlation between growth rates and oleic acid content can be observed for two of the *AtMYB4oe* lines presented here, as well as a minor reduction on the levels of C18:2 + C18:3. Additional studies on the identification of FAD2/FAD3 encoding genes and their activities in cardoon cell cultures are therefore required, as they would further drive the characterization of fatty acids accumulation in the frame of biorefinery.

While plant regeneration protocols from undifferentiated cell cultures (also from friable calluses) were successful for many different—including non-model—species (reviewed recently by [96]), cardoon or other *Cynara* species have proven recalcitrant to regeneration [97]. Cell culture-based biofactories allow for the overcoming of the difficulties of obtaining whole transgenic plants from cardoon and related species; moreover, their use is not affected by the strict genetically modified organism (GMO) legislation of several countries. However, the setup of plant regeneration protocols from wild-type and transgenic cardoon cell cultures would be of outstanding interest for translating the biochemical/physiological improvements developed in cell cultures. This would allow for their evaluation in field, in the attempt to overcome several agronomic drawbacks by which this species is otherwise notoriously affected [37,38].We are currently testing the described cardoon cell cultures potential in pilot bioreactors, with the aim of improving yields and optimizing production process; interestingly, we have so far also confirmed that in such scale-up approaches, *AtMYB4oe* lines show a significant higher productivity than WT lines (L. Langellotti—University of Naples Federico II, personal communication, October 2021). Our findings suggest that *AtMYB4oe* lines represent a valid solution to provide a fast and continuous supply of biomass with high uniformity in yields and quality. Further investigation related to life cycle assessment (LCA) is also being carried on, in order to correctly evaluate the potential of cardoon cell cultures in the frame of biorefinery.

## 4. Materials and Methods

### 4.1. Plant Material and Growth

Undifferentiated wild type (WT) friable calluses were induced from leaves of cardoon (“Spagnolo” genotype), as described in [70], with the following modifications of the callus-inducing and growth medium: Gamborg B5 agar medium including vitamins (Duchefa Biochemie, Haarlem, Netherlands, #G0209), supplied with 1 mg/L 2,4-dichlorophenoxyacetic acid (2,4-D), 1 mg/L adenine, 0.1 mg/L kinetin, 3% (*w*/*v*) sucrose, 7.5% (*w*/*v*) agar, adjusted to pH = 5.8. WT calluses were grown in the dark at 25 °C, and sub-cultured approximately every 30 days. Cell suspension cultures were started by inoculating 1.5 g of friable callus in a 250 mL Erlenmeyer flask containing 50 mL of liquid medium, and kept on a gyratory shaker at 100 rpm in the dark at 25 °C. To determine the cell growth, CVS was measured using 250 mL Erlenmeyer flasks with a graduated beak. This method has been shown to be rapid and non-destructive for the routine estimation of cell biomass, given how CVS is highly correlated with the fresh weight of cells [98]. All measures were performed on biological triplicates.

### 4.2. Construct Preparation

The coding sequence (849 bp) of *AtMYB4* (*At4g38620*) was amplified from cDNA of *Arabidopsis thaliana* mature flowers (*Col-0* ecotype) using Gateway-cloning compatible primers and Phusion HF DNA Polymerase (Thermo Fisher Scientific, Waltham, MA, USA, #F530S), and subsequently cloned in a pDONR207 vector; subcloning to pB2GW7 destination vector [99] was performed to obtain the final construct *p35S::AtMYB4*. BP and LR cloning reactions were performed according to the Gateway Cloning manual (Thermo Fisher Scientific). *A. tumefaciens* electro-competent cells (strains AGL1 and EHA105) were transformed with 100 ng of plasmid and recombinant clones were used for transformation of cardoon cells. Primers used for the construct preparation are listed in Table S6.

### 4.3. Cardoon Cell Suspension Transformation and Selection

Cell suspensions of WT cardoon were set in 250 mL sterile flasks, and kept in the dark at 25 °C under gentle agitation until the start of exponential growth (5 days); next, 5 mL of cell suspension and 10 mL of fresh liquid medium were added to a 100 mL sterile flask, and agitated for five additional days. Meanwhile, a single colony of recombinant *Agrobacterium* was used to start a 50 mL inoculum in LB medium, which was grown at 28 °C with 200 rpm shaking until OD_600_ = 0.7–0.9 (approximately 48 h), then pelleted (15 min at 2000× *g*) and re-suspended in Gamborg B5 liquid medium to a final OD_600_ = 0.80–0.85. Co-cultivation was performed adding 1 mL of *Agrobacterium* cells to each cell suspension in the presence of 400 µM acetosyringone (Sigma Aldrich, Darmstadt, Germany, #D134406), leaving flasks in the dark at 25 °C in gentle agitation for 48 h. Afterwards, pelleted cells were washed three times with fresh Gamborg B5 medium supplied with 200 mg/L CFX and 10 mg/L PPT, before being spread on sterile filter paper to remove the liquid excess; cells were then moved to the solid selective medium containing CFX and PPT. After 30 days, emerging resistant calluses were individually transferred to new plates and separately sub-cultured approximately every 21 days to fresh selective medium. PCR was used to confirm the presence of the transgene and to evaluate residual *Agrobacterium* contamination using AGL1 and EHA105 colonies as positive controls [100]. Primers used for the selection of transgenic lines are listed in Table S6.

### 4.4. Quantification of Total Phenols

Total phenolic content of the samples was determined via Folin–Ciocalteu’s method using gallic acid as a standard, as reported [101]. 1 mL of methanol/water (80:20 *v*/*v*) was added to 10 mg of freeze-dried sample before sonication (10 min), and vials were mixed in an orbital shaker overnight in the dark; after centrifugation (20 min, 13,000× *g*), 20 µL of each extract was added to 1.58 mL of deionized water and 100 µL of Folin–Ciocalteu’s reagent/water solution (1:9 *v*/*v*, Sigma Aldrich, #F9252), incubated for 8 min, then 300 µL of a 20% (*w*/*v*) Na_2_CO_3_ aqueous solution was added, and the samples were incubated in the dark for 2 h at room temperature. Measurements of absorbance at 765 nm were carried out against a reagent blank without extract in a microplate reader Infinite 200 Pro (Tecan, www.tecan.it). Total phenolic content was calculated based on a calibration curve (R^2^ = 0.99) from the gallic acid standard (Sigma Aldrich, # 398225), over the 0.004–0.25 mg/mL range. The results were expressed as mg of the Gallic Acid Equivalent (GAE) per g of DW. Measurements were carried out in triplicates. Student’s *t*-tests were run to identify significant differences among samples (*p* < 0.01).

### 4.5. Quantification of Total Lignin Content

The amount of total soluble lignin was determined by spectrophotometric measurement following derivatization with TGA, adapting a previously described method [102]. All steps were carried out at room temperature unless specified. A 60 mg freeze-dried sample was washed twice with 6 mL of phosphate buffer (100 mM K_2_HPO_4_/KH_2_PO_4,_ pH = 7.8 in water, with 0.5% Triton X-100), the tubes placed in an orbital shaker (30 min), and spun (20 min, 3200× *g*). The pellet was washed three times for 20 min in 100% methanol, and the resulting structural biomass (SBM) was dried at 80 °C (12–16 h). Three aliquots of 2 mg of SBM were mixed with 1.5 mL 2N HCl and 0.3 mL TGA (Sigma Aldrich, T3758). After incubation at 95 °C (4 h), samples were centrifuged (10 min, 13,000× *g*), and pellets washed three times with distilled water (10 min, 13,000× *g*), before incubation with 1 mL of 0.5N NaOH for 18 h in the dark. After centrifugation (15 min, 13,000× *g*), the resulting supernatant was mixed to 0.3 mL of 37% *w*/*w* HCl, and samples were incubated at 4 °C (4 h) before centrifugation (10 min, 13,000× *g*). The pellet was solubilized in 1 mL of 0.5N NaOH, and the absorbance was read at 280 nm. Lignin quantification was calculated based on a calibration curve (R^2^ = 0.99) obtained following the same steps with commercial lignin (Sigma Aldrich, #370959), over the 10^−1^–10^−4^ mg/mL range. Student’s *t*-tests were run to identify significant differences among samples (*p* < 0.01).

### 4.6. Saccharification and Evaluation of Glucose Content of Cell Walls

The saccharification yields were tested according to a previously described protocol [103], with the minor modifications reported here. 20 mg of untreated freeze-dried samples were dissolved for 5 min at 50 °C in 0.9 mL of acetic acid buffer solution (pH = 4.8), and then subjected to hydrolysis with 100 µL of a cellulase mix from *Trichoderma reesei* (Celluclast^®^ 1.5 L—Sigma Aldrich, #C2730), measuring the saccharification yield over a 48-h time interval. The enzymatic activity of the cellulase mix was 0.023 filter paper units (FPU)/mL. For each time point, an aliquot of 20 µL was used as substrate to indirectly measure glucose concentration; this was done by measuring the absorbance at 405 nm (A_405_) of the oxidized ABTS (Sigma Aldrich, #11112422001), which results from the activities of glucose oxidase (GOD, TCI Europe, Zwijndrecht, Belgium, #TCIAG0050, www.tcichemicals.com) and peroxidase (POD, Sigma Aldrich, #P6782). GOD/POD reactions and measurements of A_405_ were carried out at 37 °C in a microplate spectrophotometer Infinite 200 Pro (Tecan, Männedorf, Switzerland). Glucose content was calculated based on a calibration curve (R^2^ = 0.97) obtained following the same steps with D-glucose standards (Sigma Aldrich, #G8270), over the 20–200 µM range. Measurements were carried out in triplicates.

### 4.7. Extraction of Polyphenols

The ultrasound-assisted extraction was performed as previously reported [22]. A total of 3 g of the freeze-dried sample were extracted with 30 mL of ethanol/water (50:50 *v*/*v*) by sonication at room temperature for 30 min, centrifuged at 4 °C (10 min, 4000× *g*), filtered through 0.45 µm nylon membranes, and then used for high-resolution mass spectrometry analysis and antioxidant activity assays. 

### 4.8. Antioxidant Activity Determination 

The antioxidant activities of the polyphenolic extracts were tested with three independent strategies: DPPH, ABTS, and FRAP assays [22]. For each assay, determinations were performed in triplicates and results are expressed as Trolox^®^-equivalent antioxidant capacity (TEAC, mmol Trolox^®^-equivalents/kg of DW). ABTS in the crystallized diammonium salt form (CAS #30931-67-0) and DPPH (CAS #1898-66-4) were supplied from Merck. TPTZ (2,4,6-Tris(2-pyridyl)-s-triazine, CAS #3682-35-7) and ferric chloride (CAS #7705-08-0) used for FRAP assay were provided by Merck (Darmstadt, Germany). DPPH assay: 1 mL of DPPH solution (100 µM in methanol) was added to 0.2 mL of polyphenolic extract, and the decrease in absorbance of the resulting solution was monitored at 517 nm after 10 min; for the ABTS assay, 44 µL of aqueous 2.45 mM K_2_S_2_O_8_ were added to 7 mM aqueous ABTS solution and incubated in the dark at room temperature (23 °C) for 12 h. After dilution (1:88) with ethanol, the absorbance at 734 nm (A_734_) of this ABTS working solution was A_734_ = 0.700 ± 0.050. The assay was performed by adding 0.1 mL of filtered and suitably diluted sample to 1 mL of ABTS working solution, and the A_734_ was monitored after 2.5 min; FRAP assay: FRAP reagent contained a solution of 10 µM Tetrazolium Red (TPTZ) in 40 µM HCl, 20 µM of aqueous FeCl_3_ and acetate buffer (300 µM, pH = 3.6) at 1:1:10 (*v*/*v*/*v*). The FRAP reagent (300 µL) and sample solutions (10 µL) were mixed, and the absorbance was monitored at 593 nm after 10 min. 

### 4.9. UHPLC-HRMS Analysis of Polyphenols

The qualitative and quantitative analysis of polyphenols extracted from cardoon cell cultures was performed via High-Resolution Mass-Spectroscopy using an Ultra-High-Pressure Liquid Chromatograph Dionex UltiMate 3000 (UHPLC, Thermo Fisher Scientific), coupled with a Q-Exactive Orbitrap mass spectrometer (Thermo Fisher Scientific). The parameters were set as previously reported [22]. Chromatographic separation of polyphenolics was achieved at 25 °C with a Kinetex 2.6 µm Biphenyl (100 × 2.1 mm, Phenomenex, Torrance, CA, USA) column, with a mobile phase composed of eluent A (0.1% formic acid in water *v*/*v*) and eluent B (0.1% formic acid in methanol *v*/*v*). The gradient for elution was: 0–1.3 min 5% B, 1.3–9.3 min 5–100% B, 9.3–11.3 min 100% B, 11.3–13.3 min 100–5% B, and 13.3–20 min 5% B. The flow rate was 0.2 mL/min, and the injection volume was 2 µL. The mass spectrometer was operated in the negative ion mode (ESI-) setting for two scan events (Full ion MS and All ion fragmentation, AIF) for all compounds of interest. Full scan data were acquired setting a resolving power of 35,000 full width at half maximum (FWHM) at m/z 200. The key parameters were as follows: spray voltage −2.8 kV, sheath gas flow rate 35 arbitrary units, auxiliary-gas flow rate, 10 arbitrary units, capillary temperature 310 °C, auxiliary gas heater temperature 350 °C, S-lens RF level 50. For the scan event of AIF, the resolving power was set at 17.500 FWHM, the collision energies were 10, 20, and 45 eV, and the scan range was m/z 80–1200. Data acquisition and processing were performed with Quan/Qual Browser Xcalibur software, v. 3.1.66.10 (Thermo Fisher Scientific). 

### 4.10. Oil Extraction 

An adaptation of the solvent extraction previously described [104] was used in this study. Freeze-dried samples were extracted with hexane (1:10 *w*:*v*) for 24 h; the solution was filtered through paper under reduced pressure, and concentrated to remove the solvent in a Rotavapor RE 120 (Büchi, Essen, Germany). The final weight of the extract was recorded.

### 4.11. Fatty Acids Analysis 

Fatty acid methyl esters (FAMEs) were prepared according to previous reports [105], using 300 mg of grounded freeze-dried samples (3 replicates/sample). FAMEs were then analyzed on an Agilent 8860 GC system (Agilent Technologies, Santa Clara, CA, USA) equipped with autosampler and Flame Ionization Detector (FID). Separation was performed on an Agilent J&W DB-FastFame column (30 m × 0.25 mm × 0.25 μm). Adopted analytical conditions were as follows: carrier gas, hydrogen (10 psi, constant pressure mode); inlet, split mode (split ratio 64:1); inlet temperature 250 °C; oven, 80 °C (0.5 min), 40 °C/min to 165 °C (1 min), 4 °C/min to 230 °C (4 min); FID, 260 °C, Hydrogen (35 mL/min), Air (350 mL/min), Make-up gas 20 mL/min; and injection volume, 1 μL. Fatty acid methyl esters were identified by comparison of their retention times with those of reference standards (Supelco 37 Component FAME mix, 47885-U, Merck). Results were expressed as percentages of total fatty acid methyl esters.

### 4.12. RNA Extraction, cDNA Preparation and Expression Analysis via q-PCR

For RNA extraction, 200 mg of callus were ground under liquid nitrogen, lysed/homogenized with 1 mL of TRIzol (Thermo Fisher Scientific, #15596026), and, after addition of 0.2 mL of Phenol:Chloroform:Isoamyl-alcohol (25:24:1) and centrifugation (10 min, 13,000× *g*), the RNA was precipitated from the aqueous phase with 1 volume of isopropanol. Total RNA was treated using the Ambion TURBO DNA-free DNase (Thermo Fisher Scientific, #AM1907), and then reverse transcribed to cDNA using the High-Capacity cDNA Reverse Transcription Kit (Thermo Fisher Scientific, #4368814), according to the manufacturers’ instructions. q-PCR was performed on technical triplicates in 20 µL reactions, using the iQ5 real-time PCR detection system (Bio-Rad, Hercules, CA, USA) and 250 nM of each primer with SYBR Green PCR Master Mix (Bio-Rad, #175-5121). The q-PCR protocol used was: (95 °C 3 min) + 40 × [(95 °C 10 s) + (60 °C 30 s)], testing the melt curve over the 60 °C–95 °C range with +0.5 °C increments. The cDNA were standardized relative to the level of two different validated internal housekeeping genes: *ELF1a/Ccrd_012208* [106] and *ACT7-like/Ccrd_013113*. Relative gene expression analysis was calculated with the 2^−ΔΔCt^ method, using the Bio-Rad CFX Maestro software v. 4.0 (Bio-Rad). Student’s *t*-tests were run for each experiment to identify significant differences among samples (*p* < 0.01). Primers used in the experiments are listed in Table S6.

### 4.13. RNA-Seq, Read Mapping, Gene Expression Evaluation, GOEA Analysis and TF Prediction

Total RNAs were extracted in triplicates from four experimental groups: one WT, and three independent transgenic lines carrying the construct *p35S::AtMYB4* (*AtMYB4oe* L1, L18 and L24), for a total of 12 samples. Preliminary analysis confirmed that all samples passed the quality control (A_260_/A_280_ = 2.10, total RNA amount > 10 µg/sample and RNA integrity number > 9). Sequencing libraries were prepared according to the manufacturer’s instructions using the Illumina TruSeq RNA Sample Prep kit, and sequenced on a NovaSeq 6000 Sequencing System (Illumina, San Diego, CA, USA) to obtain 15–22 M 150 bp paired-end reads per sample. In order to preserve the longest high-quality parts of reads, trimming was performed on raw sequencing data via BBDuk software (JGI), setting the minimum length to 35 bp and the quality score to 35. The Spliced Transcripts Alignment to a Reference (STAR) software [107] was used to align the reads to the *Cynara cardunculus* reference genome (version CcrdV1, Accession number GCA_001531365), and FPKM (fragments per kilobase of exon model per million reads mapped) values were calculated for all of the genes in order to obtain expression quantification in the 12 samples. The count matrix was filtered with the HTSfilter package [108] to eliminate the genes that created uninformative signal. The identification of the differentially expressed genes (DEGs) was performed with the package edgeR [109], setting the threshold for significance of False Discovery Rate (FDR, Benjamini–Hochberg correction to *p*-value) to FDR ≤ 0.05. Data validation of Fold change (FC) values obtained by RNA-seq analysis was performed via q-PCR on 10 different sequences selected among upregulated and downregulated DEGs (q-PCR conditions as described above), and the correlation coefficient was calculated from the comparison of log_2_FC values obtained with the two methods. An informative functional interpretation of the DEGs lists was derived from a Gene Ontology Enrichment Analysis (GOEA), carried out using AgriGO v.2.0 [110] and REVIGO [111]. Visualization of enriched GO terms was performed using CirGO [112]. Amino acid sequences of DEGs were submitted to the PlantTFDB webtool (http://planttfdb.gao-lab.org/, last accessed 4 November 2021) to identify genes encoding putative transcription factors among the DEGs [113]. The Kyoto Encyclopedia of Genes and Genomes (KEGG) database (www.genome.jp/kegg, last accessed 4 November 2021) was used to annotate DEGs in metabolic pathway of interest.

## 5. Conclusions

We have shown that the protocol we set up allows the stable genetic transformation of cardoon cells. The *AtMYB4oe* lines presented in this study proved to be valuable tools for their use in bioreactors. The main advantages of these lines are represented by their faster growth rate and improved accessibility of the biomass to enzymatic degradation, due to the reduction in lignin content, which also implies an easier extractability of compounds of interest, as well as an interesting modification in their nutraceutical value. The development of this technique represents a significant step towards the industrial use of cardoon cell cultures, which can be further improved targeting specific metabolic pathways of interest; two examples would be represented by targeting other MYB transcription factors involved in the production of specialized metabolites, or by the alteration of the activity of biosynthetic genes for fatty acids, both in the frame of gain or loss-of-function genetic approaches. Moreover, further exploration of the generated RNA-seq data could provide useful to further support molecular analyses of primary and specialized metabolic pathways of cardoon cell cultures. Finally, in order to evaluate whether the use of cardoon cells for biorefinery is energetically and economically sustainable, further studies on large-scale production are being conducted. 

## Figures and Tables

**Table 1:** Biochemical characterization of WT and *AtMYB4oe* lines. Polyphenols content detected by HRMS-Orbitrap: values are expressed in ppm = µg/g dry weight, DW. 5-iFQA = 5-isoferuloyl quinic acid; 1,5-DiCQA = 1,5-dicaffeoyl quinic acid (cynarin); 3,4-DiCQA = 3,4-dicaffeoyl quinic acid; 5-FQA = 5-feruloyl quinic acid; 3-FQA = 3-feruloyl quinic acid; 3-CQA = 3-caffeoyl quinic acid (chlorogenic acid, CGA). Antioxidant activities were measured via 2,2-diphenyl-1-picrylhydrazyl (DPPH), 2,2’-azino-bis 3-ethylbenzothiazoline-6-sulfonic acid (ABTS) and Ferric Reducing Antioxidant Power (FRAP) assays. Values are expressed as TEAC: Trolox^®^-equivalent antioxidant capacity mmol Trolox/kg DW. Each value shown represents the mean values ± S.D. of three biological and two technical replicates. Fatty acids content was detected by GC-FID. Values are expressed as % of total; SFA = saturated fatty acids; MUFA = monounsaturated fatty acids; PUFA = polyunsaturated fatty acids. Different letters denote a significant difference among lines through analysis of variance (ANOVA). Statistical significance was defined as *p* < 0.05, using the Tukey’s post hoc test for mean separation.

		WT	L1	L18	L24
**Polyphenols (µg/g DW)**	5-iFQA	21.50 ± 1.33a	15.41 ± 0.43b	0.11 ± 0.04c	4.26 ± 0.12d
	1,5-DiCQA (cynarin)	3345.79 ± 112.23a	532.70 ± 11.55b	2.50 ± 0.81c	352.98 ± 21.71d
	3,4-DiCQA	3152.41 ± 26.67a	443.88 ± 12.43b	103.58 ± 8.23c	1615.65 ± 34.56d
	5-FQA	98.87 ± 11.34a	5.56 ± 0.43b	0.83 ± 0.011c	76.44 ± 11.34d
	3-FQA	3.09 ± 0.55a	2.71 ± 0.91b	0.10 ± 0.01c	10.20 ± 0.23d
	3-CQA (CGA)	319.38 ± 22.45a	424.66 ± 11.32b	97.28 ± 3.56c	513.48 ± 13.57d
	*p*-coumaric acid	3.50 ± 0.02a	3.75 ± 0.91a	1.50 ± 0.01b	3.85 ± 0.02a
	Quercetin	3.35 ± 0.02a	4.30 ± 0.02b	0.55 ± 0.01c	4.35 ± 0.02b
	Quercetin-glucoside	4.00 ± 0.65a	5.25 ± 1.23b	1.25 ± 0.03c	5.40 ± 0.34b
	Kaempferol	3.55 ± 0.65a	4.30 ± 0.91b	1.05 ± 0.03c	4.55 ± 0.83d
	Kaempferol-3-O-glucoside	1.15 ± 0.02a	1.50 ± 0.03b	0.40 ± 0.04c	1.60 ± 0.04b
	Naringin	1.20 ± 0.01a	1.75 ± 0.01b	0.41 ± 0.03c	1.800.03b
	Luteolin	1.50 ± 0.03a	2.35 ± 0.02b	0.60 ± 0.04c	2.55 ± 0.04d
	Myricetin	2.55 ± 0.34a	2.96 ± 0.32b	0.73 ± 0.11c	3.04 ± 0.12b
	Apigenin	0.20 ± 0.03a	0.25 ± 0.02b	0.01 ± 0.003c	0.30 ± 0.01d
	Total polyphenols	6962.05a	1451.33b	210.92c	2600.46d
**Antiox. activity (TEAC)**	DPPH	83.7 ± 0.61a	22.17 ± 0.42b	16.96 ± 0.63c	35.43 ± 5.82d
	ABTS	71.33 ± 0.20a	19.16 ± 0.07b	12.54 ± 0.26c	23.61 ± 0.22d
	FRAP	66.81 ± 0.98a	30.84 ± 0.21b	11.09 ± 0.08c	27.90 ± 0.54d
**Oil %**		11.38	8.24	10.35	7.25
**Fatty acids (%)**	Palmitic (C16:0)	22.29 ± 1.12ab	21.71 ± 0.58a	24.06 ± 0.35b	19.46 ± 0.38c
	Stearic (C18:0)	2.85 ± 0.31ab	3.14 ± 0.54ab	2.29 ± 0.07a	3.73 ± 0.26b
	Oleic (C18:1)	3.43 ± 1.91a	10.30 ± 0.56b	3.49 ± 0.27a	12.35 ± 0.16c
	Linoleic (C18:2)	19.38 ± 3.74a	45.82 ± 0.85b	41.04 ± 0.61c	41.34 ± 0.39c
	Linolenic (C18:3)	42.60 ± 6.34a	10.16 ± 0.38b	20.73 ± 0.74c	14.46 ± 0.06d
	Arachidic (C20:0)	0.80 ± 0.08a	0.88 ± 0.01a	0.84 ± 0.05a	1.04 ± 0.05b
	Lignoceric (C24:0)	2.42 ± 0.30a	3.26 ± 1.59a	2.41 ± 0.58a	2.40 ± 0.68a
	Nervonic (C24:1)	1.23 ± 0.14a	0.18 ± 0.03b	0.52 ± 0.47ab	0.66 ± 0.12ab
	Total SFA%	30.54 ± 0.78ab	30.29 ± 0.76a	32.07 ± 0.43b	28.00 ± 0.10c
	Total MUFA %	4.65 ± 1.777a	10.48 ± 0.30b	4.02 ± 0.21a	13.01 ± 0.28c
	Total PUFA %	61.98 ± 2.88a	55.98 ± 0.80b	61.77 ± 0.41a	55.81 ± 0.42b
	Others %	2.83	3.25	2.14	3.18

**Table 2:** Genes coding for transcription factors identified among the core-DEGs.

	Number of Core-DEGs	
TF Family	Downregulated	Upregulated
AP2	1	3
bHLH	7	11
bZIP	5	4
C2H2	1	6
C3H	2	-
Dof	3	1
ERF	2	6
FAR1	1	2
G2-like	1	3
GRAS	3	7
HB-other	2	-
HSF	1	1
LBD	3	4
MIKC_MADS	4	2
M-type MADS	2	2
MYB	1	4
MYB_related	6	5
NAC	14	3
NF-YA	1	-
Nin-like	1	-
S1Fa-like	1	-
TALE	2	3
TCP	1	-
Trihelix	1	1
WOX	2	-
WRKY	9	6
ZF-HD	1	1

## Data Availability

All RNA-seq files are available from the NCBI GEO database (accession number GSE185693).

## Supplementary Materials

Available online at https://www.mdpi.com/article/10.3390/ijms222111978/s1.
