# Supplementary material for: Towards a Cardoon (Cynara cardunculus var. altilis)-Based Biorefinery: A Case Study of Improved Cell Cultures via Genetic Modulation of the Phenylpropanoid Pathway
Source: Int J Mol Sci. 2021 Nov 5;22(21):11978. doi: 10.3390/ijms222111978 (PMC8584892; doi:10.3390/ijms222111978)
Supplement: Supplementary file 1 [file ijms-22-11978-s001.zip › Supplementary Figures and Tables - REVIEWED.pdf]

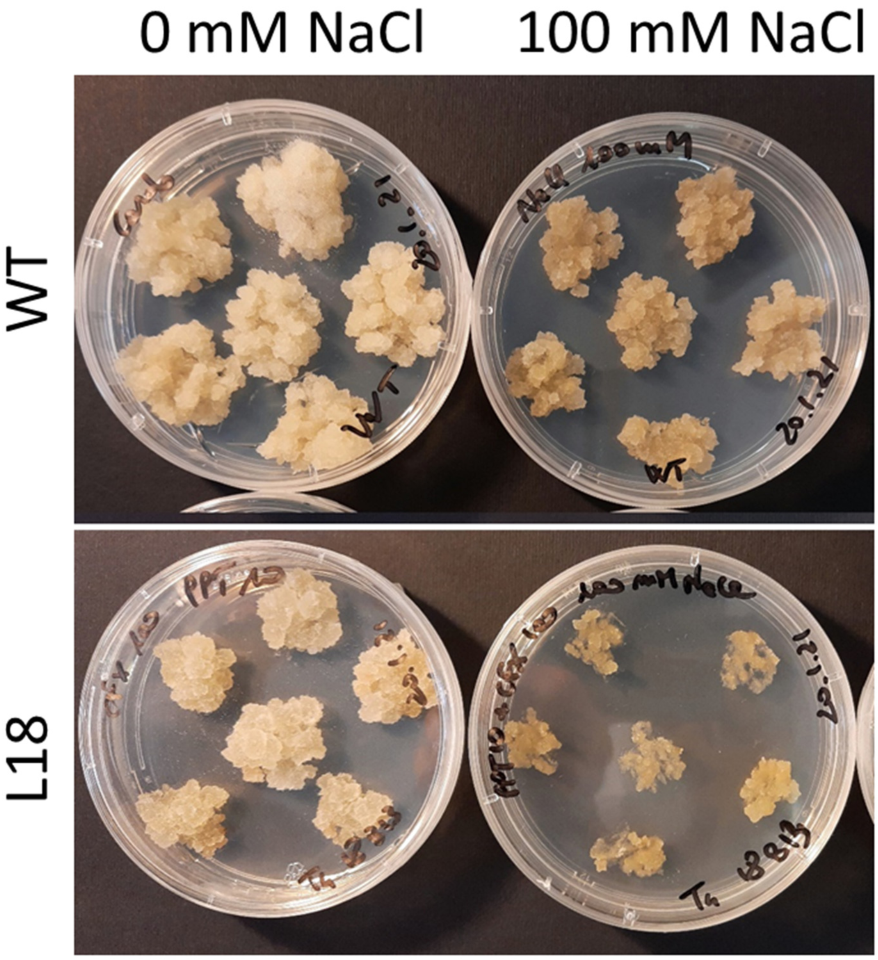

Figure S1 – Growth under salt stress conditions: WT and *AtMYB4oe* (L18) calluses grown on 0 or 100 mM NaCl-medium for 21 days.

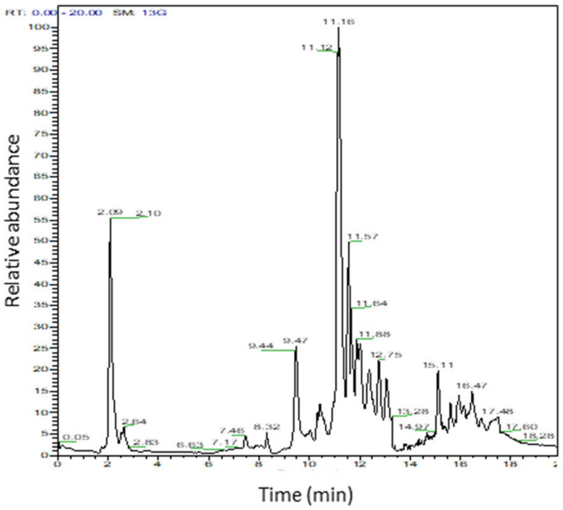

Compounds	tr	theoretical mass [M-H]⁻	experimental mass [M-H]-	accuracy (D ppm)
5 i FQA	11,86	367,10345	367,10309	-0,98
1,5-DiCQA	11,64	515,11950	515,11957	0,14
3,4-DiCQA	11,21	515,11950	515,11969	0,37
5-FQA	10,82	367,10345	367,10315	-0,82
3-FQA	9,75	367,10345	367,10335	-0,27
3-CQA	8,30	353,08798	353,08783	-0,42
Coumaric acid	9,58	163,03917	163,03912	-0,31
Quercetin-glucoside	10,21	463,08835	463,08838	0,06
Myricetin	10,61	317,03009	317,03027	0,57
Kaempferol-3-O-glucoside	10,65	447,09195	447,09326	2,93
Naringin	12,00	579,17193	579,17126	-1,16
Vitexin	9,92	431,09837	431,09818	-0,44
Quercetin	11,28	301,03549	301,03510	-1,30
Diosmin	11,38	607,16699	607,16724	0,41
Luteolin	11,54	285,04062	285,04028	-1,19
Kaempferol	11,89	285,04062	285,04025	-1,30
Naringenin	12,05	271,06110	271,06107	-0,11
Apigenin	12,14	269,04555	269,04544	-0,41

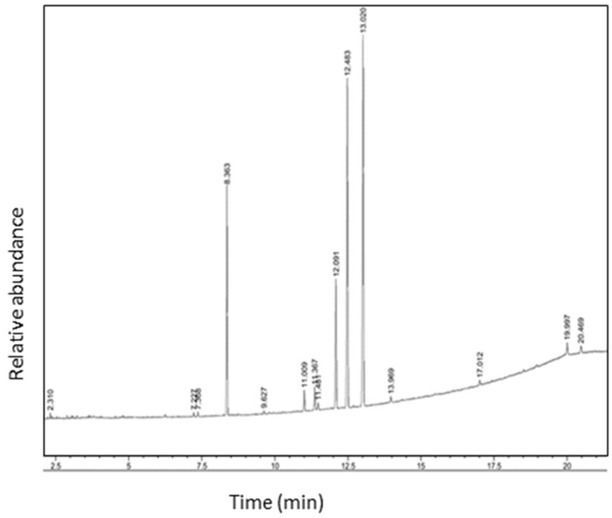

FAME	RT
unkn	2.31
MeC14:0	6.25
MeC15:0	7.22
unkn	7.37
MeC16:0	8.36
MeC17:0	9.62
MeC18:0	11.01
MeC18:1c n9	11.36
unkn	11.47
MeC18:2 c n6 (LA)	12.09
MeC 19:0 (IS)	*12.48*
MeC18:3 n3 (ALA)	13.02
MeC20:0	13.96
MeC22:0	17.01
MeC24:0	19.99
MeC24:1	20.46

Figure S2 – Biochemical profiles of phenols and fatty acids: Typical chromatograms observed for the extracts of cardoon cell lines analyzed in this study and the mass specifications of the compounds of interest relative to phenolic compounds (separation via UHPLC, upper row) and typical chromatogram and peak identification for fatty acids (separation via GC, lower row).

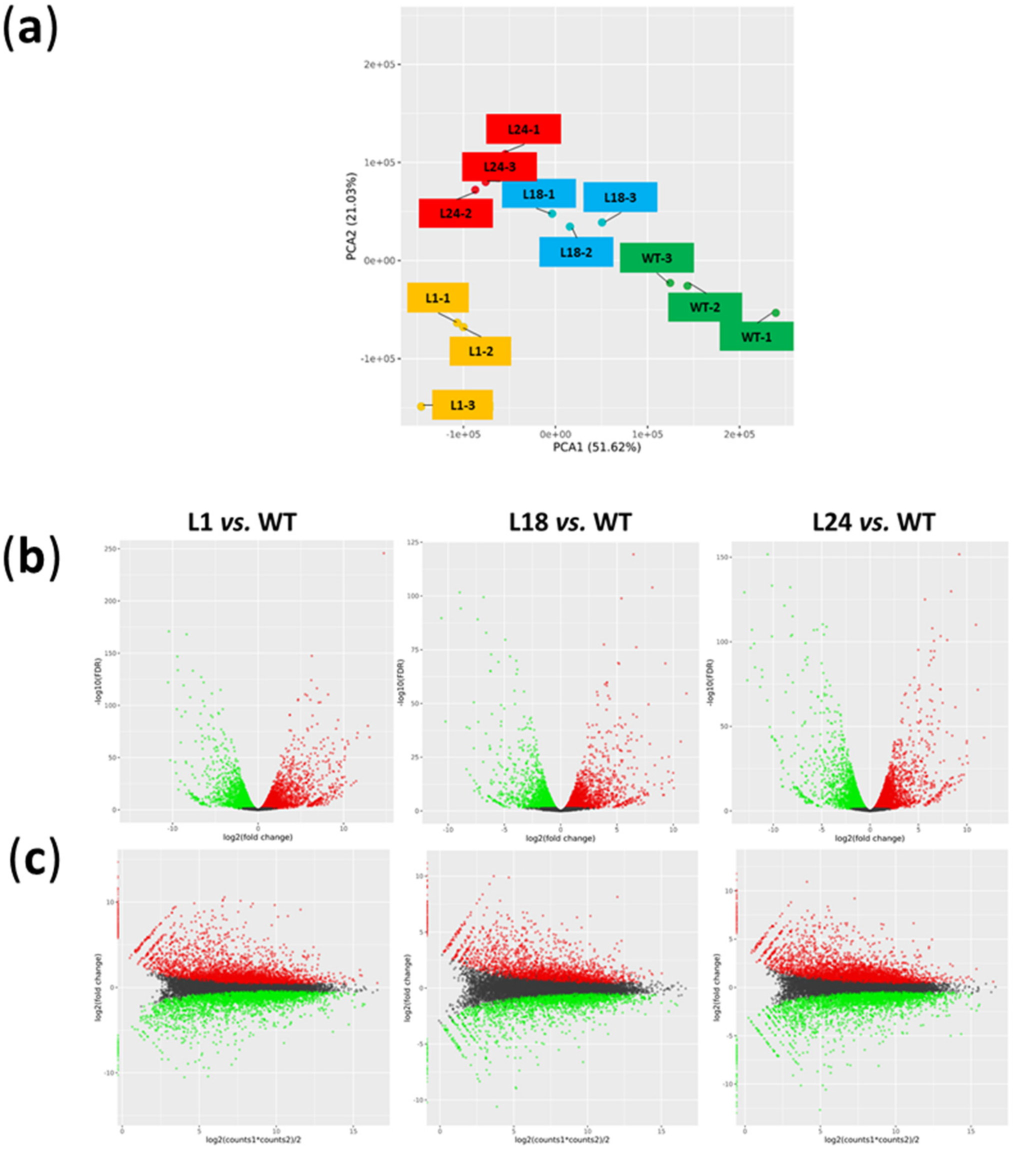

Figure S3 - Preliminary analyses on RNA-seq dataset: (**a**) Principal Component Analysis (PCA) of the 12 samples used in RNA-seq of WT and *AtMYB4oe* lines (L1, L18 and L24). For the first two PC calculated (PCA1 and PCA2) the percentage of explained variance of the dataset is visualized; (**b**) Volcano plots and (**c**) MA-plots of the differentially expressed genes - DEGs (FDR<0,05) identified from the comparison L1 vs. WT, L18 vs. WT and L24 vs. WT (green = upregulated, red = downregulated, grey = not significant).

Gene	*AtMYB4oe* line	log2(FC RT-qPCR)	log2(FC RNA-seq)
Ccrd_010165	L1	-1.84	-2.55
Ccrd_010165	L18	-1.89	-2.05
Ccrd_010165	L24	-2.25	-2.41
Ccrd_015556	L1	-0.79	-1.60
Ccrd_015556	L18	-2.00	-2.09
Ccrd_015556	L24	-1.56	-2.03
Ccrd_015561	L1	-6.64	-4.81
Ccrd_015561	L18	-2.32	-3.92
Ccrd_015561	L24	-5.06	-4.88
Ccrd_004659	L1	-7.97	-6.66
Ccrd_004659	L18	-4.06	-4.92
Ccrd_004659	L24	-8.97	-7.99
Ccrd_019107	L1	-5.64	-2.62
Ccrd_019107	L18	-8.97	-7.71
Ccrd_019107	L24	-3.47	-2.60
Ccrd_010818	L1	-2.94	-2.78
Ccrd_010818	L18	-2.91	-2.44
Ccrd_010818	L24	-4.06	-4.95
Ccrd_024577	L1	2.40	2.96
Ccrd_024577	L18	4.06	2.39
Ccrd_024577	L24	0.77	2.14
Ccrd_000418	L1	3.02	5.01
Ccrd_000418	L18	8.73	8.14
Ccrd_000418	L24	4.50	6.63
Ccrd_016332	L1	1.74	1.81
Ccrd_016332	L18	5.16	1.70
Ccrd_016332	L24	3.66	1.76

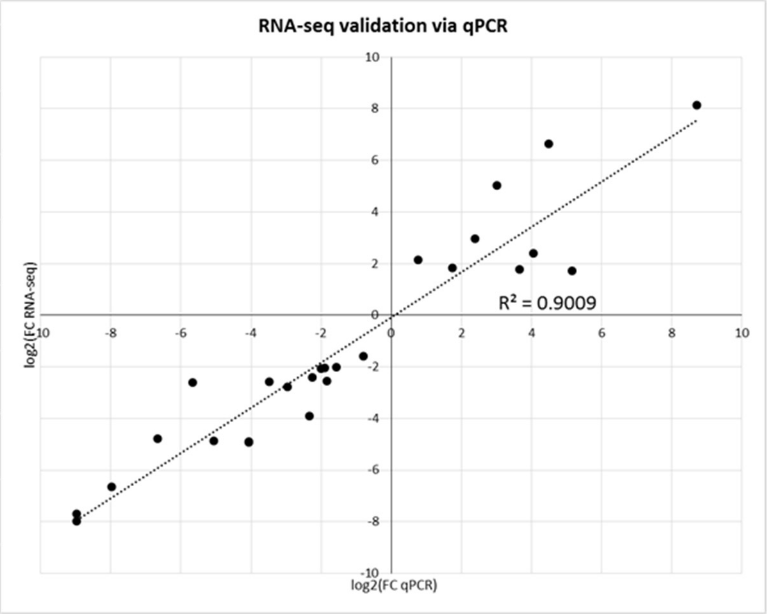

Figure S4 – Validation of RNA-seq expression levels via RT-qPCR.

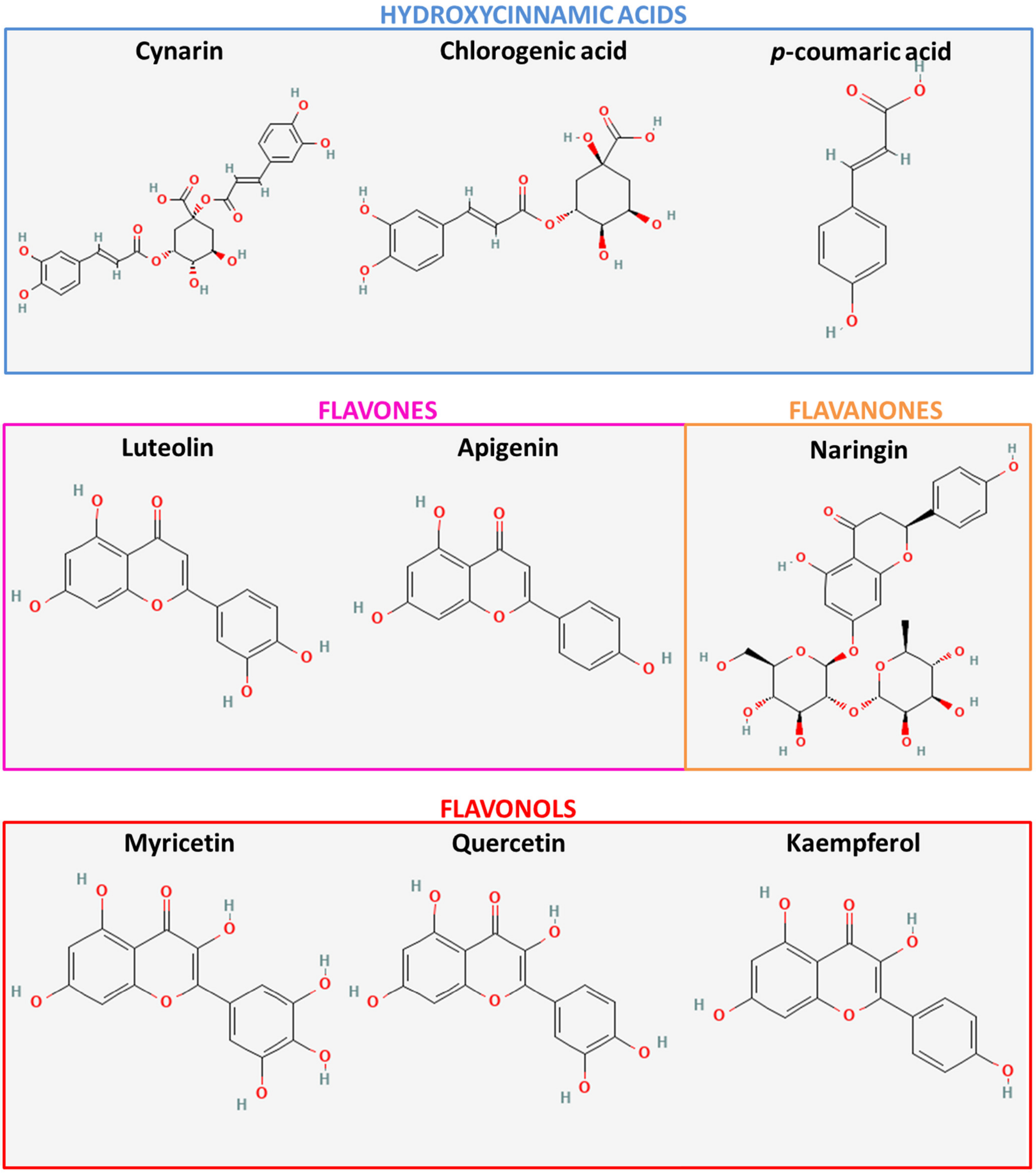

Figure S5 – Molecular structures of the main phenolic compounds identified in WT and transgenic cardoon lines.
