# Supplementary material for: Towards a Cardoon (Cynara cardunculus var. altilis)-Based Biorefinery: A Case Study of Improved Cell Cultures via Genetic Modulation of the Phenylpropanoid Pathway
Source: Int J Mol Sci. 2021 Nov 5;22(21):11978. doi: 10.3390/ijms222111978 (PMC8584892; doi:10.3390/ijms222111978)
Supplement: Supplementary file 1 [file ijms-22-11978-s001.zip › Table S1 - Mapping statistics of RNA-seq.pdf]

Sample	Input-reads	Unique	Multi	Unmapped	Mismatch-ratio	Assigned
WT-1	16584326	15752067 (94.98%)	360469 (2.17%)	471790 (2.84%)	0.31%	14216737 (85.72%)
WT-2	18019521	17073937 (94.75%)	376466 (2.08%)	569118 (3.16%)	0.30%	15515203 (86.10%)
WT-3	15934457	15175361 (95.23%)	326936 (2.05%)	432160 (2.71%)	0.30%	13807280 (86.65%)
L1-1	18391077	17361125 (94.40%)	450653 (2.45%)	579299 (3.15%)	0.31%	15628377 (84.98%)
L1-2	22792884	21547756 (94.54%)	547395 (2.40%)	697733 (3.06%)	0.30%	19509803 (85.60%)
L1-3	15190479	14299706 (94.14%)	419490 (2.76%)	471283 (3.10%)	0.31%	12736208 (83.84%)
L18-1	19349701	18350939 (94.84%)	447891 (2.31%)	550871 (2.85%)	0.30%	16543902 (85.49%)
L18-2	17394420	16502550 (94.87%)	416575 (2.39%)	475295 (2.73%)	0.30%	14903994 (85.68%)
L18-3	17483375	16624061 (95.08%)	386295 (2.21%)	473019 (2.70%)	0.29%	15125622 (86.51%)
L24-1	17761246	16774591 (94.44%)	426677 (2.40%)	559978 (3.15%)	0.32%	14895765 (83.87%)
L24-2	14924673	14115540 (94.58%)	357396 (2.39%)	451737 (3.03%)	0.32%	12505275 (83.79%)
L24-3	15477585	14573702 (94.16%)	378478 (2.44%)	525405 (3.39%)	0.33%	12784203 (82.60%)

Supporting Table S1: Mapping statistics of RNA-seq dataset.
