# Supplementary material for: Towards a Cardoon (Cynara cardunculus var. altilis)-Based Biorefinery: A Case Study of Improved Cell Cultures via Genetic Modulation of the Phenylpropanoid Pathway
Source: Int J Mol Sci. 2021 Nov 5;22(21):11978. doi: 10.3390/ijms222111978 (PMC8584892; doi:10.3390/ijms222111978)
Supplement: Supplementary file 1 [file ijms-22-11978-s001.zip › Table S4 - Expression levels of the genes involved in the phenylpropanoid pathway..pdf]

Enzyme / E.C.	Gene	log_2FC L1 *vs.* WT	log_2FC L18 *vs.* WT	log_2FC L24 *vs.* WT
PAL / 4.3.1.24	*Ccrd_007512*	-2,11	-0,96	-1,67
	Ccrd_014855	-6,26	-3,28	-4,19
C4H / 1.14.14.91	*Ccrd_003800*	-1,02	-0,78	-0,85
	Ccrd_010165	-2,55	-2,05	-2,41
4CL / 6.2.1.12	*Ccrd_015445*	*n.s.*	-0,49	-1,18
	Ccrd_015556	-1,61	-2,09	-2,03
	Ccrd_022733	-4,39	-2,94	-4,50
	Ccrd_023896	*n.s.*	*n.s.*	1,35
HCT / 2.3.1.133	*Ccrd_004326*	-0,69	*n.s.*	-1,10
	Ccrd_004546	1,22	*n.s.*	0,67
	Ccrd_009712	*n.s.*	2,20	*n.s.*
	Ccrd_009859	-3,96	-1,30	-2,80
	Ccrd_015561	-4,82	-3,93	-4,89
	Ccrd_022724	-0,59	*n.s.*	*n.s.*
C3'H / 1.14.14.96	*Ccrd_018265*	-0,82	-1,41	-1,53
	Ccrd_025643	-2,88	-2,04	-2,66
HQT / 2.3.1.99	*Ccrd_025215*	0,71	1,01	1,58
CCR / 1.2.1.44	*Ccrd_015577*	-1,98	-2,22	-2,10
CCoaOMT / 2.1.1.104	*Ccrd_004659*	-6,66	-4,92	-8,00
	Ccrd_012615	-1,73	-1,65	-1,04
	Ccrd_012620	n.s.	n.s.	0,94
F5H / 1.14.-.-	*Ccrd_005646*	-1,15	2,92	1,92
	Ccrd_018817	1,45	-0,99	n.s.
COMT / 2.1.1.68	*Ccrd_003701*	n.s.	-5,36	-3,70
	Ccrd_017109	1,65	1,55	3,17
	Ccrd_023910	-0,81	-1,25	-1,35
CAD / 1.1.1.195	*Ccrd_003953*	0,65	4,09	4,53
	Ccrd_010790	n.s.	n.s.	-0,68
	Ccrd_014274	n.s.	-3,25	-1,97
	Ccrd_020552	-1,65	3,99	n.s.
	Ccrd_020565	5,67	2,61	n.s.
	Ccrd_023113	n.s.	0,45	1,05
POD / 1.11.1.7	*Ccrd_000351*	n.s.	1,73	2,22
	Ccrd_000413	n.s.	n.s.	-2,05
	Ccrd_000415	9,56	n.s.	8,77
	Ccrd_001944	n.s.	n.s.	-1,10
	Ccrd_002344	5,07	n.s.	n.s.
	Ccrd_002896	n.s.	n.s.	3,26
	Ccrd_003014	-1,68	-2,25	-1,53
	Ccrd_004165	-3,11	-1,02	-5,81
	Ccrd_004297	4,15	2,51	2,54
	Ccrd_004475	8,94	n.s.	n.s.
	Ccrd_004478	3,78	2,01	3,19
	Ccrd_004481	2,55	n.s.	2,64
	Ccrd_004506	0,93	-1,05	1,77
	Ccrd_004507	-0,64	-1,35	n.s.
	Ccrd_004510	2,01	n.s.	2,22
	Ccrd_004513	2,20	n.s.	2,23
	Ccrd_004514	5,69	n.s.	2,83
	Ccrd_005616	8,13	n.s.	n.s.
	Ccrd_005687	5,89	3,36	4,14

Ccrd_008380	2,68	-3,39	-3,43
Ccrd_008387	1,32	n.s.	2,07
Ccrd_010480	n.s.	n.s.	-0,61
Ccrd_010635	0,98	-3,97	n.s.
Ccrd_012678	2,02	0,76	0,70
Ccrd_013040	n.s.	-2,36	n.s.
<u>*Ccrd_016862*</u>	-1,59	-1,39	-2,94
Ccrd_017982	7,29	5,19	6,50
Ccrd_018404	n.s.	n.s.	-0,72
Ccrd_018405	n.s.	n.s.	4,54
Ccrd_018407	8,45	6,76	n.s.
Ccrd_018836	2,30	3,92	1,82
<u>*Ccrd_019044*</u>	-1,42	-2,82	-2,72
Ccrd_019198	n.s.	n.s.	-0,80
Ccrd_019305	2,21	0,70	1,14
Ccrd_020667	6,73	n.s.	n.s.
Ccrd_020740	n.s.	5,92	n.s.
Ccrd_021508	1,42	n.s.	n.s.
Ccrd_022196	2,30	3,97	4,96
Ccrd_022197	-0,63	1,71	1,42
Ccrd_023159	3,40	3,04	2,69
Ccrd_024377	3,55	2,79	3,13

Supporting Table S4: Expression levels of the genes involved in the phenylpropanoid pathway. For each enzymatic step, differentially expressed genes (DEGs, FDR<0.05) between *AtMYB4oe* lines *vs.* WT are listed. Underlining indicates core-DEGs. n.s. = not significant.
