# Supplementary material for: Towards a Cardoon (Cynara cardunculus var. altilis)-Based Biorefinery: A Case Study of Improved Cell Cultures via Genetic Modulation of the Phenylpropanoid Pathway
Source: Int J Mol Sci. 2021 Nov 5;22(21):11978. doi: 10.3390/ijms222111978 (PMC8584892; doi:10.3390/ijms222111978)
Supplement: Supplementary file 1 [file ijms-22-11978-s001.zip › Table S5 - Expression levels of the genes encoding SAD and FAD2 activities.pdf]

Enzyme / E.C.	Gene	log$_2$FC L1 *vs.* WT	log$_2$FC L18 *vs.* WT	log$_2$FC L24 *vs.* WT
SAD / 1.14.19.2 - 1.14.19.11 - 1.14.19.26	*Ccrd_026332*	3.29	*n.s.*	2.49
	Ccrd_010691	*n.s.*	*n.s.*	*n.s.*
	Ccrd_010683	7.31	*n.s.*	*n.s.*
FAD2 / 1.14.19.6 - 1.14.19.22	<u>*Ccrd_009539*</u>	1.33	1.82	2.31
	Ccrd_009540	-1.53	*n.s.*	*n.s.*
	Ccrd_009531	*n.s.*	-1.44	-1.23
	Ccrd_009529	*n.s.*	*n.s.*	1.56
	Ccrd_009530	2.89	*n.s.*	2.81
	Ccrd_009537	-0.91	*n.s.*	1.23
	Ccrd_009538	*n.s.*	1.25	1.70
	Ccrd_018182	2.02	0.81	0.84
	Ccrd_012184	-2.18	1.22	*n.s.*
	Ccrd_007560	1.32	0.96	2.18
	Ccrd_007559	*n.s.*	1.38	1.81
	Ccrd_007557	*n.s.*	*n.s.*	1.77
	Ccrd_007558	*n.s.*	1.76	2.43
	Ccrd_017320	*n.s.*	*n.s.*	2.49
	Ccrd_002833	-1.79	0.98	1.82
	Ccrd_002832	3.29	*n.s.*	2.49
	Ccrd_002834	*n.s.*	*n.s.*	*n.s.*
	<u>*Ccrd_002831*</u>	2.30	1.37	2.52
	<u>*Ccrd_002830*</u>	1.32	2.04	3.08

Supporting Table S5: Expression levels of the genes encoding SAD and FAD2 activities involved in the biosynthesis of oleic acid. For each enzymatic step, differentially expressed genes (DEGs, FDR<0.05) between *AtMYB4oe* lines *vs.* WT are listed. Underlining indicates core-DEGs. n.s. = not significant.
