# Supplementary material for: Towards a Cardoon (Cynara cardunculus var. altilis)-Based Biorefinery: A Case Study of Improved Cell Cultures via Genetic Modulation of the Phenylpropanoid Pathway
Source: Int J Mol Sci. 2021 Nov 5;22(21):11978. doi: 10.3390/ijms222111978 (PMC8584892; doi:10.3390/ijms222111978)
Supplement: Supplementary file 1 [file ijms-22-11978-s001.zip › Table S6 - Primers.pdf]

**Gateway cloning of *AtMYB4* CDS**		
AtMYB4	fw	GGGGACAAGTTTGTACAAAAAAGCAGGCTTAATGGGAAGGTCACCGTGC
	rev	GGGGACCACTTTGTACAAGAAAGCTGGGTTTTATTTCATCTCCAAGCTTCGAAAGCC
**Selection of transgenic lines and expression analysis of *AtMYB4***		
AtMYB4	fw	GCAAGGGGAGTGACATGAGC
	rev	CTCCAAGCTTCGAAAGCCCA
**Checking Agrobacterium residual contamination of resistant calluses**		
virD2	fw	ATGCCCGATCGAGCTCAAGT
	rev	CCTGACCCAAACATCTCGGCTGCCCA
**Expression analyses**		
Ccrd_012208/ELF1a	fw	TCCCAGGCTGATTGTGCTGTCCTTATTAT
	rev	ATGCTCACGGGTCTGACCATCCTTA
Ccrd_013113/ACT7-like	fw	GGCGTCCTTCTTCTCCGAGAT
	rev	CATCACCAGCAAACCCAGCC
Ccrd_012103/ PAL	fw	GGGTGATGGAGAGCATGAGC
	rev	GTGCCGTTGCCAAAGATTCC
Ccrd_010165 / C4H	fw	TCATCCCAATTTTGAGGCCGT
	rev	CCATGCTTTTTGTGCTTCCCAT
Ccrd_015556 / 4CL	fw	TTCAAGTAGCACCAGCCGAG
	rev	ATGCAACTGGAACCTCTCCG
Ccrd_015561 / HCT	fw	GAGCTCCGGAAACTCATCCC
	rev	TTGCATCCCAACTCCCAGTG
Ccrd_004659 / CCoAOMT	fw	AAACGAGCGTGTATCCGAGG
	rev	GTCCTTCATCTGCCGACGTT
Ccrd_024577 / NADPH dehydrogenase	fw	AATTGGGATAGCGCCGTCT
	rev	GGAAGCTTTGCGAAGGGAAC
Ccrd_000418 / xyloglucan endoglucosylase	fw	GCCGGAGCCAATCAGTATCT
	rev	AGATGAAGCAATTTCGCCCA
Ccrd_010818 / KRP7-like	fw	ACTTCACCTCACCGTTAGAAGC
	rev	TGCAAGGAGTTTCCGCAGTT
Ccrd_019107 / MADS-TF	fw	TGAAACAACCACCTCAAACTCAC
	rev	GCTGGTACTCACGGGTTGAA
Ccrd_016332 / AP2-TF	fw	CCGACACACTAGCTTAGCACA
	rev	ATTGCAACCACACAACCCTTT

Supporting Table S6 – Primers used in this study.
